# Large Genomic Fragment Deletions and Insertions in Mouse Using CRISPR/Cas9

**DOI:** 10.1371/journal.pone.0120396

**Published:** 2015-03-24

**Authors:** Luqing Zhang, Ruirui Jia, Norberto J. Palange, Achim Cchitvsanzwhoh Satheka, Jacques Togo, Yao An, Mabwi Humphrey, Luying Ban, Yan Ji, Honghong Jin, Xuechao Feng, Yaowu Zheng

**Affiliations:** 1 Transgenic Research Center, School of Life Sciences, Northeast Normal University, Changchun, China; 2 Key Laboratory of Molecular Epigenetics of Ministry of Education, Northeast Normal University, Changchun, China; Ohio State University Comprehensive Cancer Center, UNITED STATES

## Abstract

ZFN, TALENs and CRISPR/Cas9 system have been used to generate point mutations and large fragment deletions and insertions in genomic modifications. CRISPR/Cas9 system is the most flexible and fast developing technology that has been extensively used to make mutations in all kinds of organisms. However, the most mutations reported up to date are small insertions and deletions. In this report, CRISPR/Cas9 system was used to make large DNA fragment deletions and insertions, including entire *Dip2a* gene deletion, about 65kb in size, and β-galactosidase (lacZ) reporter gene insertion of larger than 5kb in mouse. About 11.8% (11/93) are positive for 65kb deletion from transfected and diluted ES clones. High targeting efficiencies in ES cells were also achieved with G418 selection, 46.2% (12/26) and 73.1% (19/26) for left and right arms respectively. Targeted large fragment deletion efficiency is about 21.4% of live pups or 6.0% of injected embryos. Targeted insertion of lacZ reporter with NEO cassette showed 27.1% (13/48) of targeting rate by ES cell transfection and 11.1% (2/18) by direct zygote injection. The procedures have bypassed *in vitro* transcription by directly co-injection of zygotes or co-transfection of embryonic stem cells with circular plasmid DNA. The methods are technically easy, time saving, and cost effective in generating mouse models and will certainly facilitate gene function studies.

## Introduction

Genetically modified mice represent a powerful tool for dichotomizing gene functions [1,2]. Traditionally, mice carrying targeted mutations are generated by homologous recombination [3]. The technology takes the advantage of cultured embryonic stem cells and chimera generation. The procedures are tedious, less cost-effective and time-consuming. Zinc-finger nucleases (ZFN) [4], transcription activator-like effector nucleases (TALENs) [5,6] and CRISPR/Cas9 system are recently developed technologies in genomic editing. Engineered ZFNs, TALENs and CRISPR/Cas9 have been successfully used to direct site-specific cleavage and mediate error-prone non-homologous end-joining (NHEJ) or precise homologous recombination (HR) when a DONOR DNA is provided [7,8]. However, ZFN is very tedious in finding efficient DNA binding blocks which requires experiences and TALENs are time-consuming in building the DNA binding domains. Both systems use an artificial nuclease domain (FokI) to make double-stranded breaks (DSBs). CRISPR/Cas9 system is the most updated and the most easily applicable tools. In this system, genome editing is achieved through the bacterial type II clustered regularly interspaced short palindromic repeats (CRISPR) [9–11] and CRISPR-associated protein 9 (Cas9), a very efficient nuclease itself, to target interested genes and to make specific double-strand breaks (DSBs) [12]. The only required engineering is a 20nt target-complementary CRISPR RNA (crRNA) with the target DNA sequence sitting upstream of a 5’protospacer adjacent motif (PAM) site [13]. Various organisms including zebrafish [14], mouse [1], monkey [15], rat [16] and human cells have been successfully modified [17,18].

It has been a major problem for genomic editing that involves large DNA fragment insertion, deletion or replacement, where the larger the fragment, the lower the recombination efficiency [19]. This results in a fact that many reports having modified fragment size around 1kb. However in many cases, large genomic sequence changes are required, for example, deletion of gene clusters, removal of long non-coding RNAs (lncRNA) and swapping of regulatory sequences. Different technologies have been developed to tackle this problem. For examples, BAC and YAC systems have been used to target relatively large DNA fragments. But the efficiency is far from satisfactory. Recently, Xiao and coworkers have reported cleavage of genomic sequence of up to 1Mb in zebrafish by applying TALENs and CRISPR/Cas9 [20]. In this report, two circular plasmids expressing sgRNA and Cas9 were co-injected into mouse zygote to minimize the laborious and extra-careful preparation of *in vitro* transcribed Cas9 mRNA and sgRNAs [21], leading to complete deletion of the entire *Dip2a* gene, a 65kb fragment. When the same two circular plasmids were co-transfected with a DONOR plasmid containing selective marker, the 65kb-*Dip2a* fragment was successfully replaced with a NEO cassette at high frequency in ES cells. A 5.3kb fragment of lacZ reporter gene and NEO cassette was also successfully inserted into downstream of *Dip2a* promoter by both direct injection and ES cell transfection with high efficiency.

## Materials and Methods

### Animals

The Institutional Animal Care and Use Committee or Animal Experimental Ethics Committee of Northeast Normal University (NENU/IACUC) has specifically approved the entire study, the approval # is AP2013011. This study was carried out in strict accordance with the recommendations in the Guide for the Care and Use of Laboratory Animals of the National Institutes of Health as well.

129S1/SvJ, C57BL/6J, DBA/2 and CD1 mice were purchased from Vital River (A Charles River Company, Beijing, China). All animals were maintained in a clean facility in Northeast Normal University. Mice were kept in IVC cages (5 per cage) with free access of food and water, under pathogen-free conditions in a room maintained at 20°C and 50 ± 20% relative humidity, and under a 12:12-h light:dark cycles. Mice were anesthetized before sacrificing with 1% pelltobarbitalum natricum at a dose of 10 mg/kg. All animal experiments were performed using proper anesthesia before perfusion or any procedures.

### Plasmids

Plasmid pX330 (http://www.addgene.org/42230/) [18] and pRosa26-1 were obtained from Addgene. pX330 is gifted from Dr. Feng Zhang and the DTA sequence containing pRosa26-1 targeting vector is gifted from Dr. Philippe Soriano's lab. pMD18T-simple T-vector was purchased from Takara, Dalian, China. The pL253 and pL451 are from NCI-Frederick [22], pTie2-LacZ-YWZ was modified plasmid from pg50-2.11 (Gifted from Dr. Tom Seto) by Yaowu Zheng. All the restriction endonucleases and modifying enzymes were from New England Biolabs, and Fermentas USA, or Takara, Dalian, China. Oligos were synthesized by Genewiz, Suzhou, China. All the cell culture medium were purchased from Life Technology, Inc. Cell culture supplies were from Nunc, USA. Fetal bovine serum certified for ES cell culture was purchased from Hyclone, USA. Gonadotropin, Pregnant Mare Serum (PMSG, 367222) is from Calbiochem and human Chorionic Gonadotropin (hCG, C1063) is from Sigma, USA. EmbryoMax injection buffer (MR-095-10F) is from Millipore.

### pX330-sgRNAs design and expressing vector construction

A 250bp sequence containing the targeting region was submitted to CRISPR Design Tool (http://crispr.mit.edu/, Zhang Feng Lab), the sgRNAs with highest score were chosen and corresponding oligos were ordered. Oligos were re-suspended in H_2_O to 100uM final concentration. 9μl sense oligo, 9μl reverse oligo and 2μl 10× annealing buffer (100mM Tris, pH8.0, 1.5M NaCl) were mixed and boiled then cooling overnight. 1μl of 100 times diluted oligos was used to ligate with 50ng of BpiI linearized pX330 vector. Correct insertion was verified by BpiI restriction digestion and DNA sequencing using TRC-F: CAAGGCTGTTAGAGAGATAATTGGA primer. sgRNA sequences are: D2A-sgINTR1-F: CACCGAGCCCACTTGTCAATGACGT, D2A-sgINTR1-R: AAACACGTCATTGACAAGTGGGCTC; and D2A-sg3’UTR-F: CACCGTGTTAGAACATCCTCCGAAC, D2A-sg3’UTR-R: AAACGTTCGGAGGATGTTCTAACAC. sgRNA for targeted insertion is described in the results part.

### DONOR vector construction

#### Intermediate vector pL253-NEO-DTA construction

To generate a general blank targeting vector called pL253-DTA-Linker, we used polymerase chain reaction (PCR) amplified DTA fragment from pRosa26-1 and Hsv-TkpA from pL253 plasmids respectively. Primer sequences are: DTA-F: GGCCTGATCAGCCACCATGGATCCTGATGATGTTGTTGATTCTTCTAAATCTTTTGTGATGGAAAACTTTTCTTCG, and DTA-R: GGCCTCGAGTTAGAGCTTTAAATCTCTG; Hsv-TKpA-F: GGCCGTCGACAACACGGAAGGAGACAATAC, and HsvTKpA-R: GGCCGGTACCCGTGGTGACCAATACAAAAC. Gel purified products were cloned into pMD18T vector for DNA sequencing. The DTA fragment was then recovered by double digestion of pMD18T-DTA recombinant plasmid with BclI, XhoI and Hsv-Tk with SalI, KpnI that were then cloned into pL253 vector digested with BglII and KpnI. Correct insertion was confirmed by XhoI single digestion, KpnI/PstI double digestion and DNA sequencing. A 59bp sequence linker annealed with pL253-DTA-Linker-F: GGCCGGCGCGCCGTCGACATCGATTGCGGCCGCCAATTGAAGATCTGATATCCATATGC, pL253-DTA-Linker-R: TCGAGCATATGGATATCAGATCTTCAATTGGCGGCCGCAATCGATGTCGACGGCGCGCC oligos was inserted into pL253-DTA-Hsv-TKpA intermediate construct double digested with NotI/XhoI. Insertion of linker was confirmed by DNA sequencing. frt-NEO-frt-loxP was obtained by double digestion of pL451 plasmid with EcoRI/BamHI. The fragment was then inserted at BglII/MunI of the linker site to give pL253-frt-NEO-frt-loxP-DTA.

#### *Dip2a* LacZ knockin DONOR vector construction

Approximately 750bp *Dip2a* right arm was PCR amplified from C57BL/6J genomic DNA using PrimeSTAR MAX High Fidelity DNA polymerase. PCR product was SalI/EcoRV double digested and cloned into pL253-frt-NEO-frt-loxP-DTA digested with XhoI/EcoRV. *Dip2a* left arm was also PCR amplified and was cloned into pMD18T-simple vector. Primers used are: *Dip2a* Right arm F: GTCAGATATCGGATGCCCTCTGGAGG, *Dip2a* Right arm R: GTCAGTCGACCTTGCCCTCTAATCTGACA; and *Dip2a* Left arm F: GTCAGGCGCGCCAGAGGGTGGCTAATGAGTAG, *Dip2a* Left arm R: GTCGACATCGATGTCAGTCCAT*GG* *TGG*AACCGGGGCCTGCAGACC (italic indicates KOZAK sequence). All the PCR products were confirmed by sequencing and blasted against C57BL/6J sequences. LacZ fragment was obtained by NcoI/NarI double digestion of pTiez-LacZ-YWZ recombinant plasmid. The fragment was then inserted into NcoI/ClaI site of double digested pMD18T-simple-750bp-left arm. To obtain the final construct, the previous product was double digested with AscI/SalI and a 4180bp fragment corresponding to *Dip2a* left arm plus lacZ was cloned into pL253-frt-NEO-frt-loxP-3’Arm-DTA digested with AscI/SalI. EcoRV restriction digestion was performed to assess the correct insertion of *Dip2a* left arm and LacZ.

#### *Dip2aΔ65kb* DONOR vector construction

A 720bp right arm and 967bp left arm were PCR amplified respectively from C57BL/6J genomic DNA using following primers: D2A5’Arm-F: GTCGACGGATAATGCAAATTCTGGTCAAG, D2A5’Arm-R: GCGGCCGCCCACTTGTCAATGACGTAGGAAA; and D2A3’Arm-F: GATATCAACCGGCTAAAGCAGTGTTTTGG, D2A3’Arm-R: CTCGAGGGCACATAACTCAAATGCAAAGC. PCR fragments were cloned into pMD18T-simple vector first and then sequence confirmed. The right arm clone was digested with EcoRV/XhoI and cloned into EcoRV/XhoI digested pL253-frt-NEO-frt-loxP-DTA. The left arm was cloned into the final construct using SalI and NotI double digestion.

### Cell Culture and Transfection

Mouse 129×B6 ES cells were isolated according to previous reports [23,24]. Clones previously tested with good germ line transmission were used in this study. Mouse ES cells were cultured on gelatin-coated dishes using Knockout-DMEM (Invitrogen) supplemented with 15% FBS (ES cell tested, Hyclone, USA), 1×MEM nonessential amino acids (Invitrogen), 1×β-mercaptoethanol (Invitrogen), 1×penicillin and streptomycin (Invitrogen), and 1000 U/ml leukemia inhibitory factor, 1uM PD0325901 (Selleck, USA) and 3uM CHIR99021 (Selleck, USA), under 37°C and 5% CO_2_. One 35mm-dish of ES cells of approximately 80% confluence were transfected using Amaxa Nucleofector machine (Lonza, USA), program A-30, 0.2cm cuvette and Nucleofector Kit (VPH-1001). For testing promoter activity in 129×B6 F1 ES cells, hybrid CBA (CBh) promoter from pX330 and cytomegalovirus (CMV) early enhancer/chicken β actin (CAG) promoter from pCAGGS were compared to drive expression of the enhanced green fluorescent protein (EGFP). For optimization, different amount of supercoiled pCBh-EGFP-N1 plasmid were tested for nucleofection. For *Dip2aΔ65kb* DONOR free electroporation, 1.5μg of each supercoiled pX330-sgRNA were used and 300 cells per 100mm-dish were seeded and maintained for 72 hours for clone picking. For electroporation with *Dip2aΔ65kb* DONOR, 1μg of each supercoiled pX330-sgRNA and 2μg of supercoiled DONOR DNA were used. 24hs later, cells were selected with 200μg/ml G418 for 5 days. Single isolated colonies were picked and grown in duplicates on 96 well plates for screening by PCR. For *Dip2a* lacZ knockin Nucleofection, 3μg supercoiled DONOR plasmid and 3μg supercoiled pX330-sgRNA plasmid per 35mm-dish were used.

### Microinjection

Sexually emergent female F1 (B6×DBA/2) mice (4 weeks old) were superovulated by intraperitoneal injection of 5 IU PMSG followed by 5 IU hCG at an interval of 46h and mated overnight with C57BL/6J stud male mice. Zygotes were collected in M2 medium the next morning from the infundibulum region of the oviduct, digested with hyaluronidase, and transferred into the M16 medium. Microinjection was performed using an Olympus IX71 inverted microscope equipped with Narishige microinjector. Two supercoiled plasmids encoding sgRNA and Cas9 (pX330-sgRNAs) for *Dip2aΔ65kb*, or supercoiled pX330-sgRNA plus supercoiled DONOR in 1× injection buffer (each 5ng/μl) for *Dip2a* LacZ knockin were co-injected into pronuclear. All zygotes were cultured overnight to two cell stage and transferred to the pseudo-pregnant CD1 females.

### Genotyping

The screening of founder mice was performed by tail PCR and sequencing. Tail tips were digested in GNT-K buffer at 55°C overnight [25]. Then tail preps were diluted and boiled for 15 min. For *Dip2aΔ65kb*, PCR was performed with Taq DNA Polymerase (Takara Bio) and following primers: Dip2A16490F: ACCCAGAATGTTTGTGAGGCTTA, and Dip2A82061R: GTTCTCTCCAGCATAGACCTTACA. PCR genotyping yields a 600bp fragment as expected if deletion happens. The PCR products were gel-purified with a Kit (Qiagen, Germany) and sequenced. *Alpl* locus in right arm was used as PCR control (Alpl-F: ATGATCTCACCATTTTTAGTACTGG, and Alpl-R: ACCGACCTCCTTATCTGGTAGTG). For *Dip2a* LacZ knockin genotyping, a short LacZ sequence of 250bp (LacZ-F: ACCACACCTCCTGCCTGTATAAC, and LacZ-R: ACGACGGGATCATCGCGAGCCAT), was amplified. For correct recombination, primers used are: (Dip2A5’ScrF: ACCACACCTCCTGCCTGTATAAC, and LacZ5’R: ACGACGGGATCATCGCGAGCCAT; BGHpA-F: AGCTGGGGCTCGACTAGAGCTT, and Dip2A3’ScrR: CCTAGTCTGTTTCTAACAACCACT). Due to the extreme high GC% content in the 5’ Arm, we use GC buffer I (Takara) and slowdown PCR program [26]. None specific integration of plasmid backbone, DONOR plasmid or Cas9 gene were checked with following primer pairs (Amp-F: AGATGCTGAAGATCAGTTGGGTG, Amp-R: GTCATGCCATCCGTAAGATGCTT; pUC-F: CGGATCAAGAGCTACCAACTCTT, pUC-R:GCCTTATCCGGTAACTATCGTCT;Cas9-F1: AAGGTGGACGACAGCTTCTTCCA, Cas9-R1: AACAGCGACGTGGACAAGCTGTT; Cas9-F2: CAGCCAGATCCTGAAAGAACACC, Cas9-R2: CTTTCTCTGGGTAATCAGCTTGG).

### Off-target assay

Potential off-target sites (OTS) were identified using CRISPR Design Tool and 6 OTS were chosen for each sgRNA. 500bp Fragment containing OTS were amplified from founder DNA using PrimeSTAR High Fidelity enzyme. The PCR products were gel-purified and re-annealed in NEBuffer 2. 0.5μl of T7 Endonuclease I (T7ENI) were added and digested for 30min at 37°C. Products were run on 1.5% agarose gel. Detailed primer and OTS information are in S1 Table.

### LacZ staining

E10.5 embryos were fixed in 2% PFA, 0.25% Glutaraldehyde, and 0.01% NP-40 in phosphate buffered saline (PBS) at 4°C for 15 min with agitation. Fixed embryos were washed 3 times for 20min each in rinse buffer (2mM MgCl_2_, 0.02% NP-40 and 0.01% Sodium deoxycholate in PBS), and incubated in staining buffer [30mM K_3_Fe(CN)_6_, 30mM k_4_Fe(CN)_6_•3H_2_O, 2mM MgCl_2_, 0.01% Sodium deoxycholate, 0.02% NP-40 and 1mg/ml 5-bromo-4-chloro-3-indolyl-b-D-pyranoside (X-gal) in PBS ph7.4] at 37°C 6∼12hrs until an appropriate staining intensity was obtained. After staining, embryos were washed in PBS for 3×20 minutes and post-fixed in 4% PFA overnight with agitation followed with 3× wash in large volume of 1×PBS. Embryos were photographed with Olympus microscope equipped with Canon digital camera.

## Results

### Circular plasmids can mediate long range and high efficiency deletion in ES cells

Recent *in vitro* studies suggest DIP2A to be the receptor of FSTL1 and mediate numerous FSTL1 biological functions [27,28]. *Fstl1* KO (knockout) mice have shown overt phenotypes, such as hydroureter [29], septal hypercellularity and end-expiratory atelectasis [30] *et al*. Mouse *Dip2a* encompasses ∼80kb on chromosome 10 and has eight transcription variants due to the alternative splicing. Using traditional knockout techniques, only part of this gene can be removed that leaves most of the gene intact and could generate some splicing variants. To completely remove the entire gene, from the first intron to the last exon, two guide RNAs were designed using the web CRISPR Design Tool, one located in the first intron with score 88 and the other at 3’UTR of *Dip2a* gene scored 92.

**Fig 1:**
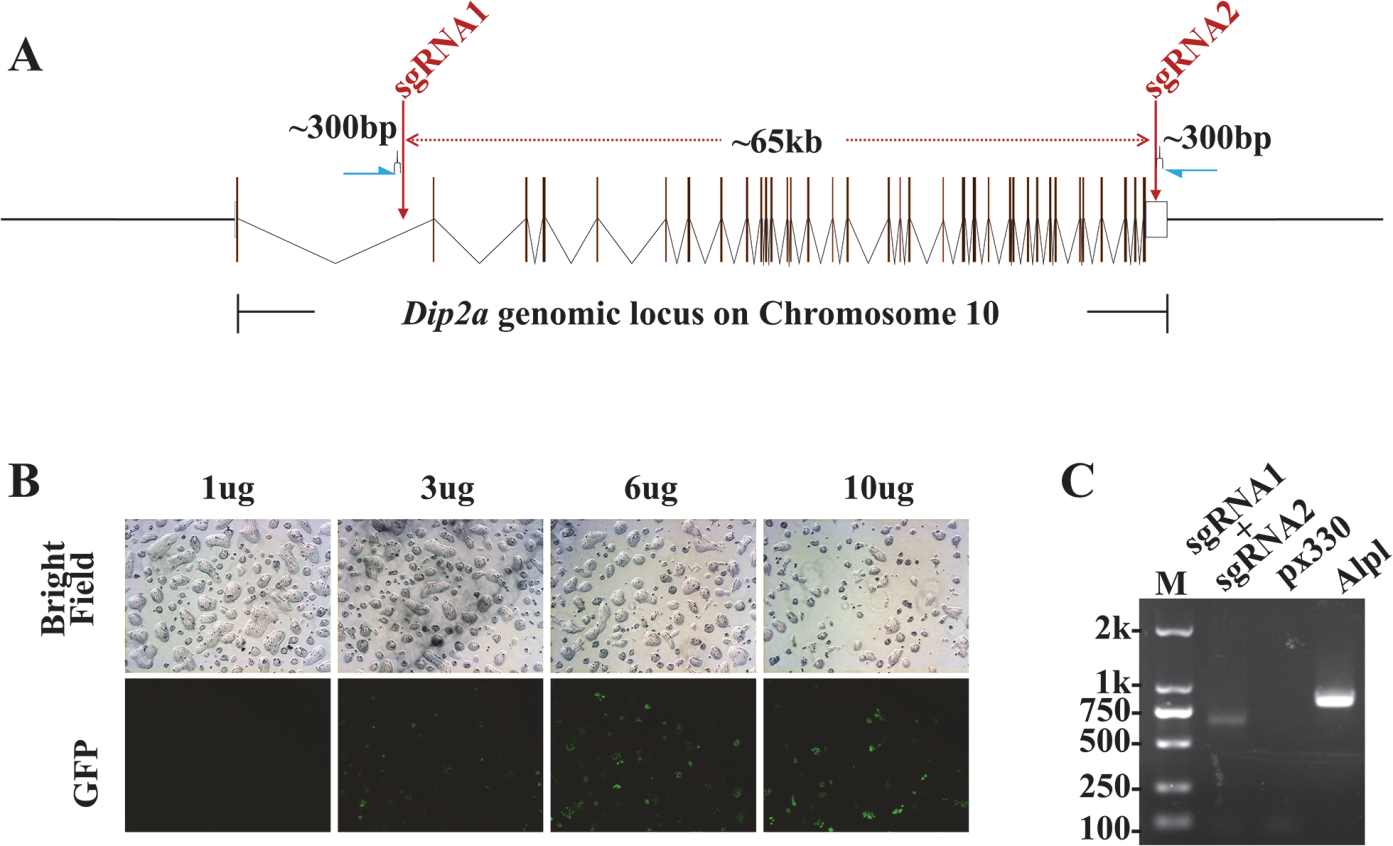
Circular plasmid mediated large genomic deletion in mES cells. (A) *Dip2a* gene genomic locus, sgRNAs cutting sites and genotyping strategy. Blue arrows indicate genotyping primers. (B) Optimization of mouse embryonic stem cell nucleofection with pCBh-EGFP-N1 plasmid. (C) Genotyping nucleofected mES pool for ∼65kb deletion.

The oligos were annealed and cloned into pX330 vector as previously described. The strategy of targeting non-coding sequence was to prevent complete disruption of the gene due to high NHEJ rate in the other chromosome in case of potential lethality of KO. The two sgRNA bearing pX330 plasmids (pX330-sgRNAs) were introduced into the ES cells by nucleofection, the most effective transfection method tested. Nucleofection of ES cells was first optimized by using supercoiled pCBh-EGFP-N1 plasmid. Using 3μg and 6μg per 6-well can achieve as high as 40% transfection efficiency based on EGFP expression. Higher amount of DNA resulted to lower cell viability and transfection efficiency (Fig. 1B). CBh promoter is as efficient as CMV promoter, however, CAG showed much weaker expression in ES cells (Data not shown). The CRISPR/Cas9-mediated cleavage in ES cells was checked by PCR 72 hours post transfection with two primers, one designed shortly before the first sgRNA cleaving site and the other shortly after the second sgRNA cleavage site (Fig. 1A, a distance of ∼300bp on each side). As predicted, correctly targeted cells (with 65kb deletions) produced a band of ∼ 600bp while no band was detected on cells transfected with empty pX330 vector alone. The faint band may (Fig. 1C) suggest that a portion of the cells are deletion mutants in large numbers of wild type ES cells, compared to the primer pairs efficiently amplifying *Alpl*. Amplification efficiency was compared between *Alpl* and homozygous *Dip2a* KO locus (S1 Fig.). To isolate pure *Dip2a* 65kb-deletion ES clones, 300 ES cells were split onto 100mm cell culture dish with feeder cells after nucleofection. Three days later, single clones were picked, dispersed and cultured in 96 well plate in duplicate. About 11.8% (11/93) of clones were positive for deletion by PCR and sequencing.

### Circular plasmid expression can induce long range homologous recombination in ES cells

**Fig 2:**
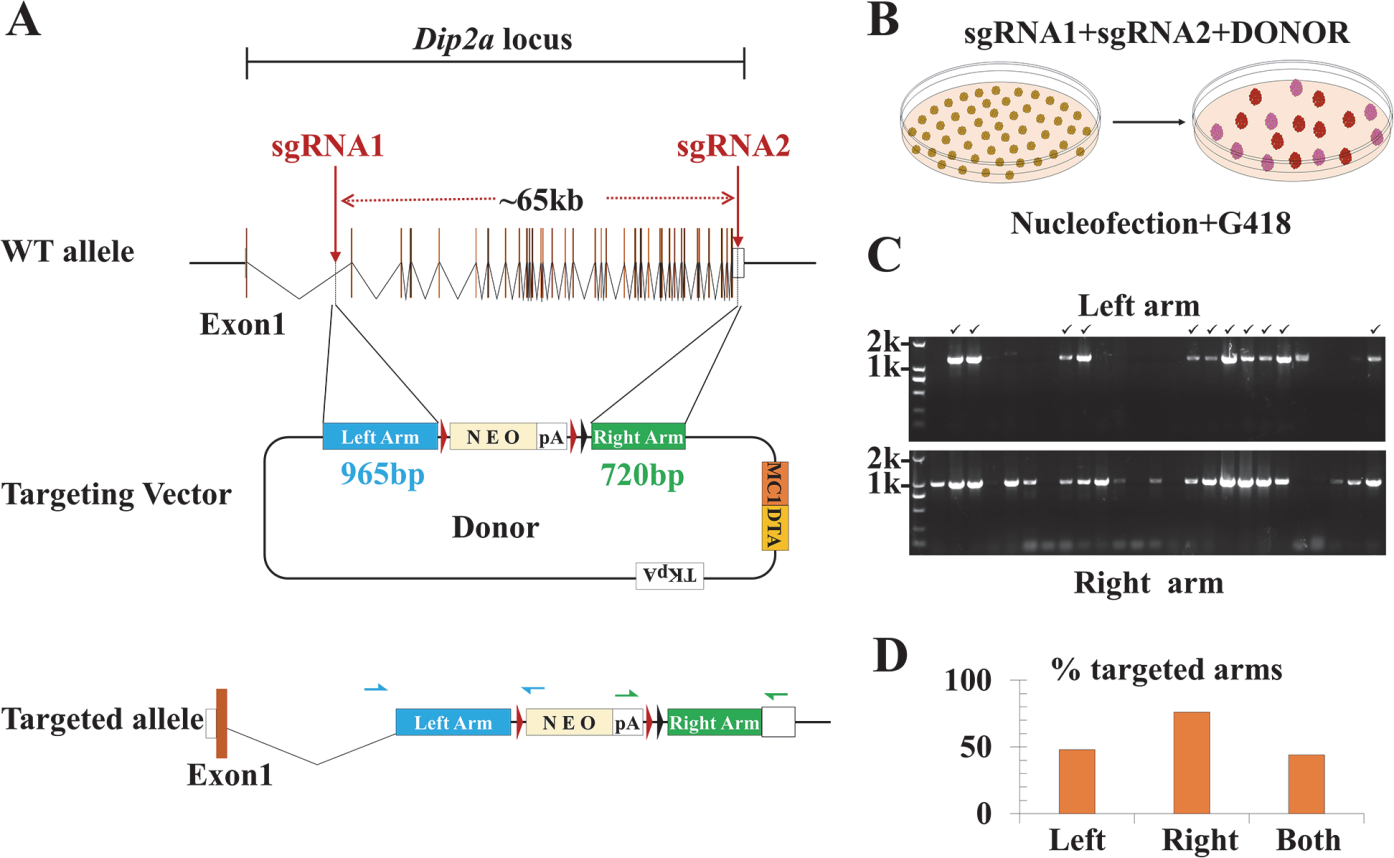
Plasmid mediated long range homologous recombination in mES cells. (A) Targeting strategy of *Dip2a* gene. The designed primer pairs for screening are indicated with blue and green. (B) Schematic illustration of nucleofection and selection. The targeted clones in red and non-targeted in pink. (C) Recombination screening of left and right arm by PCR. (D) Targeting efficiency with sgRNAs and DONOR in mES cells.

Traditional gene knockout by homologous recombination is inefficient. It requires long homologous arms, usually longer than 5kb, to produce reasonable targeting efficiency. DNA fragments of deleted or recombined are mostly 20kb or less [31]. We co-transfected two circular targeting plasmids and a DONOR vector with NEO expression cassette into ES cells and analyzed homologous recombination efficiency. The DONOR vector contains a NEO cassette flanked by two *Frt* and one *loxP* sites. This cassette is further flanked by 965bp and 720bp homology arms (Fig. 2A). Cells were selected with G418 at 200μg/ml 24h after transfection. Single colonies that have survived were checked for homologous recombination by PCR (Fig. 2C). The 65kb locus was replaced with NEO cassette with high efficiency. The targeting efficiencies are 46.2% (12/26) and 73.1% (19/26) for left and right arms respectively (Fig. 2C&D, a single sample run on another gel was not shown). The *Frt* sites flanking the NEO cassette are removable by FLP recombinase and can be changed to a *loxP* variant (loxP2272). This made further engineering possible for RMCE (recombinase mediated cassette exchange), a tool of great importance in generation of humanized mouse models.

### Circular plasmids can generate long range deletion in zygotes with high efficiency

**Fig 3:**
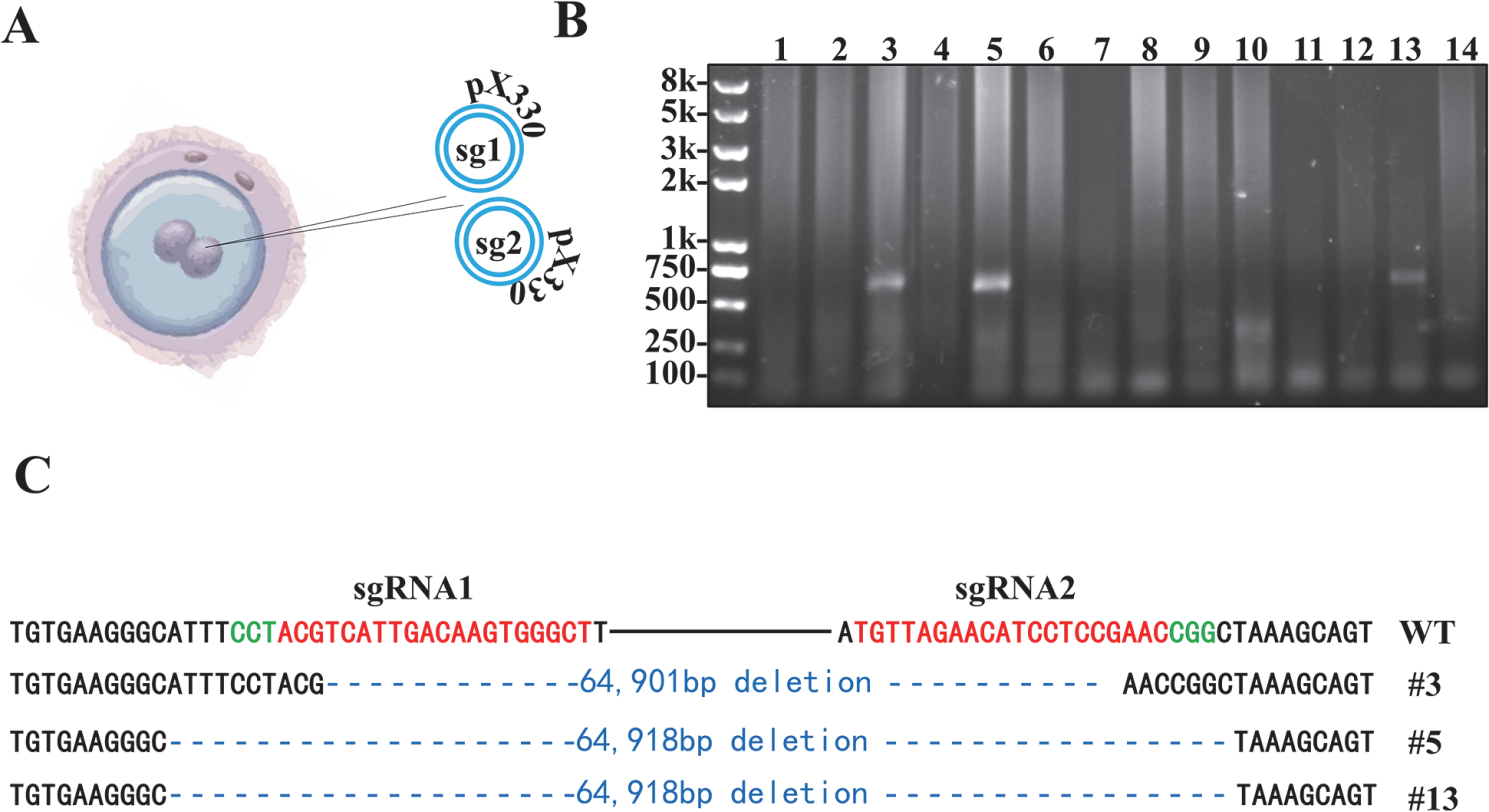
Circular plasmid mediated high efficiency deletion in zygotes. (A) Injection of two sgRNA-pX330 plasmids at 5ng/μl each into zygote. (B) Genotyping 14 pups with the same primers in Fig. 1. (C) PCR sequencing of 3 pups with deletion. The PAM sequence is highlighted in green, the targeting sites are in red and the deleted regions are in blue.

Given the successful removal of ∼65kb fragment and homology-direct repair (HDR) at ES cells level, CRISPR/Cas9 system was tested to mediate deletion of a large genomic fragment in mice by direct pronuclear injection of the same circular plasmids. Mashiko and coworkers (2013) has reported generation of mutant mice (381bp deletion) by pronuclear injection of circular plasmids expressing Cas9 and sgRNAs into mouse zygotes [21]. Up to date, the maximum reported CRISPR/Cas9-mediated deletion in mouse was 10kb by optimized sgRNA and Cas9 mRNA injection [32]. The same two circular plasmids used for ES cell transfection were used for zygote injection at 5ng/μl each. A total of 50 zygotes were injected and cultured overnight (Fig. 3A). On next day, 42 zygotes developed to two cell stage and were transferred to 3 pseudo-pregnant CD1 mice. Among 14 live pups, 3 contained targeted deletion (Fig. 3B). For further confirmation, the PCR product was gel purified and sequenced (Fig. 3C). Mutant #5 and #13 share the same deletion sequence which was confirmed to be individual events by repeated tail cutting and sequencing. All three founders were germline transmitted, with the transmission rate of 50.0% (4/8), 53.8% (7/13) and 12.5% (1/8) for #3, #5 and #13 respectively.

### CRISPR-Cas9 can mediate efficient knockin of large reporter genes in ES cells

**Fig 4:**
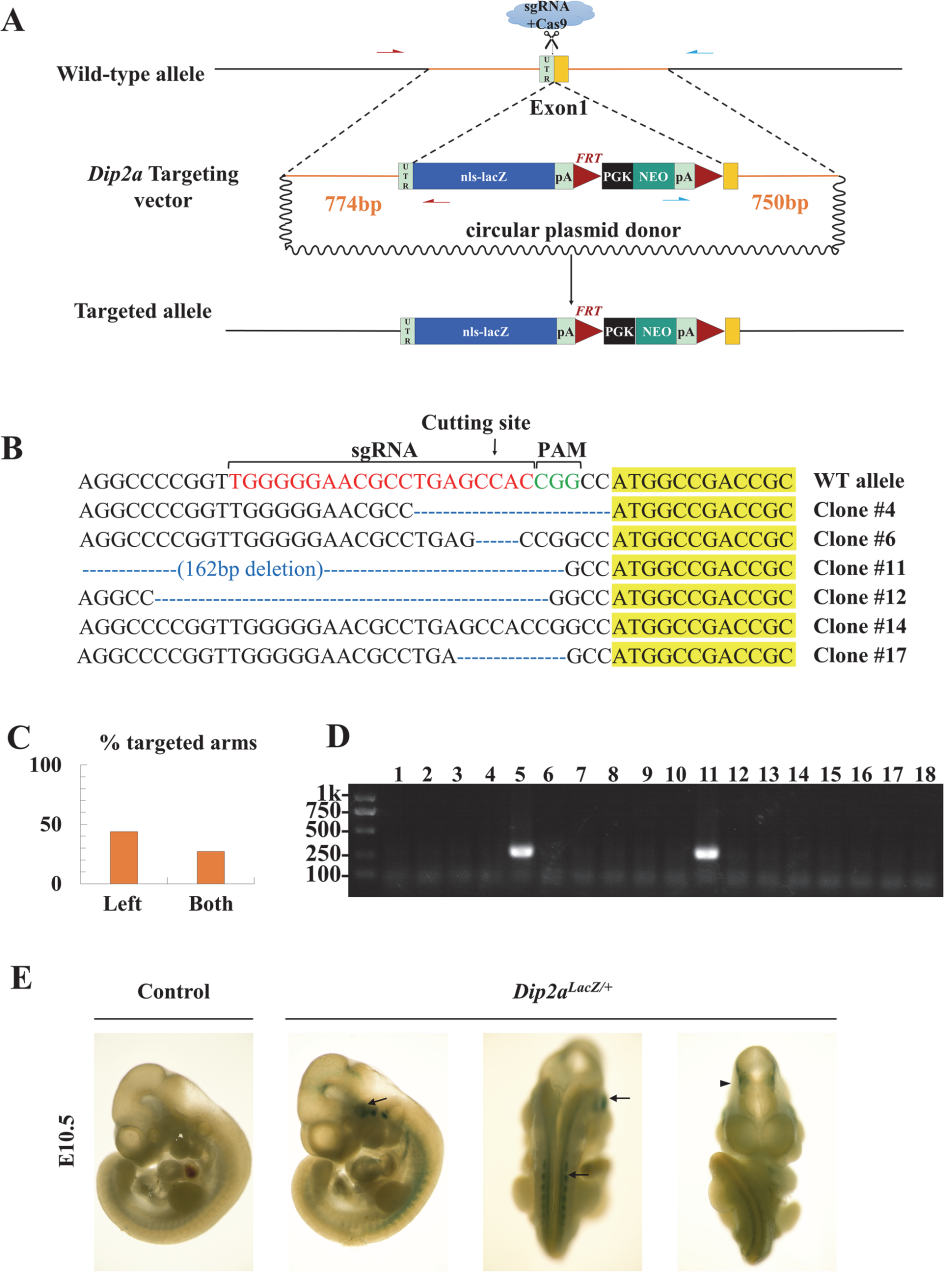
CRISPR-Cas9 mediated lacZ-NEO knockin. (A) Overview of targeting strategy. The LacZ gene with NEO selection cassette is inserted ahead of *Dip2a* ATG start codon. (B) The non-knockin alleles were PCR amplified and sequenced. CRISPR/Cas9 mediate indels are shown in detail. (C) Targeting frequency of G418 resistant mES clones. (D) Genotyping of 18 pups from direct zygotes injection with sgRNA-pX330 and DONOR plasmids. Two are positive for LacZ PCR. (E) LacZ staining of E10.5 *Dip2a* ^*LacZ/+*^ embryos. DIP2A is expressed moderately in the brain (arrowhead) and at high level in the spinal cord, dorsal root ganglion and trigeminal ganglion (arrow).

LacZ reporter mice have made huge contribution in revealing gene expression patterns and developmental studies. Expression patterns of *Dip2a* gene has never been systematically studied although important biologic functions have been suggested. To generate LacZ knockin mice, a knockin DONOR vector was constructed (Fig. 4A). A sgRNA targeting site with sequence of TGGGGGAACGCCTGAGCCAC***CGG*** was designed using web CRISPR Design Tool (http://crispr.mit.edu/) and scored 73, which is just two nucleotides ahead of start codon, the ATG site in exon 1. Top strand oligo CACCGTGGGGGAACGCCTGAGCCAC and bottom strand oligo AAACGTGGCTCAGGCGTTCCCCAC were annealed and cloned into pX330. The two plasmids, 3μg each, were nucleofected into ES cells in 35mm plate and selected with G418 at 200μg/ml for 5 days. Single colonies were transferred to 96 well plate and checked for homologous recombination by PCR. The position of primers are shown in Fig. 4A. After screening by PCR, 43.8% (21/48) were found recombined on right arm. Among them, 61.9% (13/21) had correct left arm recombination, or 27.1% (13/48) correct recombination in both arms (Fig. 4C). Correctly targeted cells were verified on the other non-knockin allele for mutations. Compared to the PCR-amplified wild type 478bp-fragment, 5 clones from the total 6 sequenced clones showed deletions from 3bp to 162bp (Fig. 4B). One clone was found intact. As expected, all the mutations were in the noncoding region and predicted to be harmless.

### CRISPR-Cas9 can mediate efficient knockin of large reporter genes in mouse

The same combinations of plasmids were subject to direct zygote injection. The DONOR plasmid and pX330-sgRNA plasmid were injected at 5ng/μl each. From 145 injected zygotes, 89 developed to two cell stage and were transferred to 6 CD1 pseudo-pregnant mice. From 18 live pups, two were found positive by LacZ PCR (Fig. 4D). Correct recombination of both left and right arms were confirmed by PCR using primer pairs indicated in Fig. 4A. All transgenic pups were germline transmitted and LacZ stained positive in embryonic stages. Fig. 4E shows the moderate expression of DIP2A in brain (arrowhead) and high expression in spinal cord, dorsal root ganglion and trigeminal ganglion (arrow).

## Discussion

Discovery and development of chimeric nucleases, such as ZFNs and TALENs, have made genetic editing easy by triggering a designer-targeted DNA DSBs that stimulate error-prone NHEJ or HDR [21,33]. Due to difficulties encountered in the process of vector design and construction, these methods have been quickly supplemented or replaced by a more simple, fast and economic CRISPR/Cas9 system. This RNA-guided DNA targeting can mediate insertions and deletions (*indels)* in mice by co-expression of Cas9 mRNA and sgRNAs [1]. However, RNA preparation requires some careful steps such as *in vitro* transcription, RNA handling and storage. In this study, only circular plasmid DNA has been used to transfect or directly inject zygotes. Large DNA fragment can be easily deleted. Mutant #5 and #13 sharing the same deletion sequence indicated deletion preference may exist. CRISPR/Cas9 plasmid construction are easy, fast and economic. With help of DONOR plasmid, high efficiency of insertion or exchange of large fragments can be achieved. Although the injection of plasmid DNA expressing sgRNA and Cas9 can give none specific transgenic integration, we did not find any such event in our founders (S2 Fig.).

Xiao and coworkers have managed a direct cleavage of approximately 1Mb in zebrafish by applying TALENs and CRISPR/Cas9 *in vitro* transcribed mRNAs [20], but to our best knowledge, no studies or reports were documented in mouse yet. Fujii and coworkers managed to delete a 10kb region in mice using CRISPR/Cas9 system [32]. However, it is still challenging to make large genomic modifications over 20kb in mice. In this study, CRISPR-Cas9 has been proved to be a feasible and simple system to manipulate large genome fragment with high efficiency. The result indicates that deletion of 65kb DNA fragment or insertion of 5.3kb DNA fragment by homologous recombination are highly efficient both by ES cell transfection and by direct injection of zygotes.

In this report, it is demonstrated that CRISPR/Cas9 can mediate efficient homologous recombination (HR) of large DNA fragment in ES cells, much more efficient than traditional gene targeting. Traditional homologous recombination targeting method could remove at most 20kb genome sequence with very low efficiency and large homology arms are required that makes PCR screening very difficult [22]. Valenzuela et al using modified BAC (Bacteria artificial chromosome) vectors achieve 70kb genomic sequence deletion in mouse ES cells, however the targeting efficiency is about 1% [34]. Generation of targeting vector is also complicated and time consuming. In this report, the homologous arms of the DONOR vector is less than 1kb and easy to amplify from genomic DNA by PCR. Therefore, generation of the targeting vector is relatively easy and fast. PCR screening of targeted allele is also much easier compared to longer arms.

LacZ reporter mice are very powerful models to dissect gene expression patterns and to study developmental events. Targeted insertion of reporter lacZ can better mimic the endogenous gene expression than traditional transgenic mice. To generate lacZ reporter mice by direct injection of circular DONOR plasmid into zygotes and homologous recombination can save time and resources. The sgRNAs were designed based on web CRISPR Design Tool (Zhang Feng Lab) and high score targets were used. A limited number of potential loci were checked for off-target effect with T7 Endonuclease I (T7ENI), but no mutations were found (S3 Fig.). To conclude, CRISPR/Cas9 system, with optimum design, can be used to manipulate large genomic DNA fragment deletion, insertion and exchange with high efficiency. The procedures used in this report are extremely easy and fast. It will certainly facilitate gene function studies and generation of better disease models.

## Supporting Information

S1 Fig The primers for amplification of *Δ65kb* and *Alpl* have similar efficiency. Genomic DNA from *Dip2a* ^*Δ65kb/Δ65kb*^ mice has been extracted and used to PCR for *Alpl* and *Δ65kb* locus for either 30 or 35 cycles.
(TIF)

S2 Fig No transgenic insertions of pX330 plasmid or DONOR DNA were detected. Genomic DNA from tail of *Dip2a* ^*Δ65kb/WT*^ and *Dip2a* ^*LacZ/WT*^ founder mice were subjected to PCR amplification for Ampicillin Resistant region (Amp), pUC replication origin (pUC), and two regions of Cas9 gene. Amp and pUC sequence exist on both DONOR and pX330 and Cas9 regions only exist on pX330 plasmid.
(TIF)

S3 Fig T7EN assay of six off-target sites (OTS) for *Dip2a* Δ65kb sgRNA1, sgRNA2 and *Dip2a* LacZ Knock-in (KI) sgRNA (see S1 Table). No obvious off target site observed.
(TIF)

S1 Table Summary of off-target sites (OTS) for each sgRNA and primers used for amplifying corresponding OTS. (XLSX)

S1 File Sequence of pL253-NEO-DTA. (DOCX)

S2 File Sequence of Dip2a-LacZ Knockin DONOR Vector. (DOCX)

S3 File Sequence of Dip2a Δ65kb DONOR vector. (DOCX)

S4 File Sequence of pTie2-LacZ-YWZ. (DOCX)

S5 File Sequence of pCBh-EGFP-N1. (DOCX)

## References

[1] Wang H, Yang H, Shivalila CS, Dawlaty MM, Cheng AW, Zhang F, et al One-step generation of mice carrying mutations in multiple genes by CRISPR/Cas-mediated genome engineering. Cell. 2013;153(4):910–918. 10.1016/j.cell.2013.04.025 23643243 PMC3969854

[2] Mizuno S, Dinh TT, Kato K, Mizuno-Iijima S, Tanimoto Y, Daitoku Y, et al Simple generation of albino C57BL/6J mice with G291T mutation in the tyrosinase gene by the CRISPR/Cas9 system. Mammalian genome: official journal of the International Mammalian Genome Society. 2014;25(7–8):327–334. 24879364 10.1007/s00335-014-9524-0

[3] Capecchi MR. Generating mice with targeted mutations. Nature medicine. 2001;7(10):1086–1090. 11590420 10.1038/nm1001-1086

[4] Cui X, Ji D, Fisher DA, Wu Y, Briner DM, Weinstein EJ. Targeted integration in rat and mouse embryos with zinc-finger nucleases. Nature biotechnology. 2011;29(1):64–67. 10.1038/nbt.1731 21151125

[5] Tesson L, Usal C, Menoret S, Leung E, Niles BJ, Remy S, et al Knockout rats generated by embryo microinjection of TALENs. Nature biotechnology. 2011;29(8):695–696. 10.1038/nbt.1940 21822240

[6] Carlson DF, Tan W, Lillico SG, Stverakova D, Proudfoot C, Christian M, et al Efficient TALEN-mediated gene knockout in livestock. Proceedings of the National Academy of Sciences of the United States of America. 2012;109(43):17382–17387. 10.1073/pnas.1211446109 23027955 PMC3491456

[7] Ramalingam S, Annaluru N, Chandrasegaran S. A CRISPR way to engineer the human genome. Genome biology. 2013;14(2):107 10.1186/gb-2013-14-2-107 23448668 PMC3663103

[8] Shen B, Zhang W, Zhang J, Zhou J, Wang J, Chen L, et al Efficient genome modification by CRISPR-Cas9 nickase with minimal off-target effects. Nature methods. 2014;11(4):399–402. 10.1038/nmeth.2857 24584192

[9] Segal DJ. Bacteria herald a new era of gene editing. eLife. 2013;2:e00563 10.7554/eLife.00563 23386979 PMC3557904

[10] Chang N, Sun C, Gao L, Zhu D, Xu X, Zhu X, et al Genome editing with RNA-guided Cas9 nuclease in zebrafish embryos. Cell research. 2013;23(4):465–472. 10.1038/cr.2013.45 23528705 PMC3616424

[11] DiCarlo JE, Norville JE, Mali P, Rios X, Aach J, Church GM. Genome engineering in Saccharomyces cerevisiae using CRISPR-Cas systems. Nucleic acids research. 2013;41(7):4336–4343. 10.1093/nar/gkt135 23460208 PMC3627607

[12] Ding Q, Regan SN, Xia Y, Oostrom LA, Cowan CA, Musunuru K. Enhanced efficiency of human pluripotent stem cell genome editing through replacing TALENs with CRISPRs. Cell stem cell. 2013;12(4):393–394. 10.1016/j.stem.2013.03.006 23561441 PMC3925309

[13] Hsu PD, Scott DA, Weinstein JA, Ran FA, Konermann S, Agarwala V, et al DNA targeting specificity of RNA-guided Cas9 nucleases. Nature biotechnology. 2013;31(9):827–832. 10.1038/nbt.2647 23873081 PMC3969858

[14] Hwang WY, Fu YF, Reyon D, Maeder ML, Tsai SQ, Sander JD, et al Efficient genome editing in zebrafish using a CRISPR-Cas system. Nature biotechnology. 2013;31(3):227–229. 10.1038/nbt.2501 23360964 PMC3686313

[15] Niu Y, Shen B, Cui Y, Chen Y, Wang J, Wang L, et al Generation of gene-modified cynomolgus monkey via Cas9/RNA-mediated gene targeting in one-cell embryos. Cell. 2014;156(4):836–843. 10.1016/j.cell.2014.01.027 24486104

[16] Ma Y, Zhang X, Shen B, Lu Y, Chen W, Ma J, et al Generating rats with conditional alleles using CRISPR/Cas9. Cell research. 2014;24(1):122–125. 10.1038/cr.2013.157 24296780 PMC3879705

[17] Mali P, Yang L, Esvelt KM, Aach J, Guell M, DiCarlo JE, et al RNA-guided human genome engineering via Cas9. Science. 2013;339(6121):823–826. 10.1126/science.1232033 23287722 PMC3712628

[18] Cong L, Ran FA, Cox D, Lin SL, Barretto R, Habib N, et al Multiplex Genome Engineering Using CRISPR/Cas Systems. Science. 2013;339(6121):819–823. 10.1126/science.1231143 23287718 PMC3795411

[19] Canver MC, Bauer DE, Dass A, Yien YY, Chung J, Masuda T, et al Characterization of genomic deletion efficiency mediated by clustered regularly interspaced palindromic repeats (CRISPR)/Cas9 nuclease system in mammalian cells. The Journal of biological chemistry. 2014;289(31):21312–21324. 10.1074/jbc.M114.564625 24907273 PMC4118095

[20] Xiao A, Wang Z, Hu Y, Wu Y, Luo Z, Yang Z, et al Chromosomal deletions and inversions mediated by TALENs and CRISPR/Cas in zebrafish. Nucleic acids research. 2013;41(14):e141 10.1093/nar/gkt464 23748566 PMC3737551

[21] Mashiko D, Fujihara Y, Satouh Y, Miyata H, Isotani A, Ikawa M. Generation of mutant mice by pronuclear injection of circular plasmid expressing Cas9 and single guided RNA. Scientific reports. 2013;3:3355 10.1038/srep03355 24284873 PMC3842082

[22] Liu P, Jenkins NA, Copeland NG. A highly efficient recombineering-based method for generating conditional knockout mutations. Genome research. 2003;13(3):476–484. 12618378 10.1101/gr.749203 PMC430283

[23] Pieters T, Haenebalcke L, Hochepied T, D'Hont J, Haigh JJ, van Roy F, et al Efficient and user-friendly pluripotin-based derivation of mouse embryonic stem cells. Stem cell reviews. 2012;8(3):768–778. 10.1007/s12015-011-9323-x 22011883 PMC3412084

[24] Ye S, Tan L, Yang R, Fang B, Qu S, Schulze EN, et al Pleiotropy of glycogen synthase kinase-3 inhibition by CHIR99021 promotes self-renewal of embryonic stem cells from refractory mouse strains. PloS one. 2012;7(4):e35892 10.1371/journal.pone.0035892 22540008 PMC3335080

[25] Malumbres M, Mangues R, Ferrer N, Lu S, Pellicer A. Isolation of high molecular weight DNA for reliable genotyping of transgenic mice. BioTechniques. 1997;22(6):1114–1119. 9187761 10.2144/97226st03

[26] Frey UH, Bachmann HS, Peters J, Siffert W. PCR-amplification of GC-rich regions: 'slowdown PCR'. Nature protocols. 2008;3(8):1312–1317. 10.1038/nprot.2008.112 18714299

[27] Ouchi N, Asaumi Y, Ohashi K, Higuchi A, Sono-Romanelli S, Oshima Y, et al DIP2A functions as a FSTL1 receptor. The Journal of biological chemistry. 2010;285(10):7127–7134. 10.1074/jbc.M109.069468 20054002 PMC2844162

[28] Tanaka M, Murakami K, Ozaki S, Imura Y, Tong XP, Watanabe T, et al DIP2 disco-interacting protein 2 homolog A (Drosophila) is a candidate receptor for follistatin-related protein/follistatin-like 1—analysis of their binding with TGF-beta superfamily proteins. The FEBS journal. 2010;277(20):4278–4289. 10.1111/j.1742-4658.2010.07816.x 20860622

[29] Xu J, Qi X, Gong J, Yu M, Zhang F, Sha H, et al Fstl1 antagonizes BMP signaling and regulates ureter development. PloS one. 2012;7(4):e32554 10.1371/journal.pone.0032554 22485132 PMC3317656

[30] Geng Y, Dong Y, Yu M, Zhang L, Yan X, Sun J, et al Follistatin-like 1 (Fstl1) is a bone morphogenetic protein (BMP) 4 signaling antagonist in controlling mouse lung development. Proceedings of the National Academy of Sciences of the United States of America. 2011;108(17):7058–7063. 10.1073/pnas.1007293108 21482757 PMC3084141

[31] Cheah SS, Behringer RR. Contemporary gene targeting strategies for the novice. Molecular biotechnology. 2001;19(3):297–304. 11721625 10.1385/MB:19:3:297

[32] Fujii W, Kawasaki K, Sugiura K, Naito K. Efficient generation of large-scale genome-modified mice using gRNA and CAS9 endonuclease. Nucleic acids research. 2013;41(20):e187 10.1093/nar/gkt772 23997119 PMC3814358

[33] Miller JC, Holmes MC, Wang J, Guschin DY, Lee YL, Rupniewski I, et al An improved zinc-finger nuclease architecture for highly specific genome editing. Nature biotechnology. 2007;25(7):778–785. 17603475 10.1038/nbt1319

[34] Valenzuela DM, Murphy AJ, Frendewey D, Gale NW, Economides AN, Auerbach W, et al High-throughput engineering of the mouse genome coupled with high-resolution expression analysis. Nature biotechnology. 2003;21(6):652–659. 12730667 10.1038/nbt822

