# Supplementary figures and images for: Large Genomic Fragment Deletions and Insertions in Mouse Using CRISPR/Cas9
Source: PLoS One. 2015 Mar 24;10(3):e0120396. doi: 10.1371/journal.pone.0120396 (PMC4372442; doi:10.1371/journal.pone.0120396)

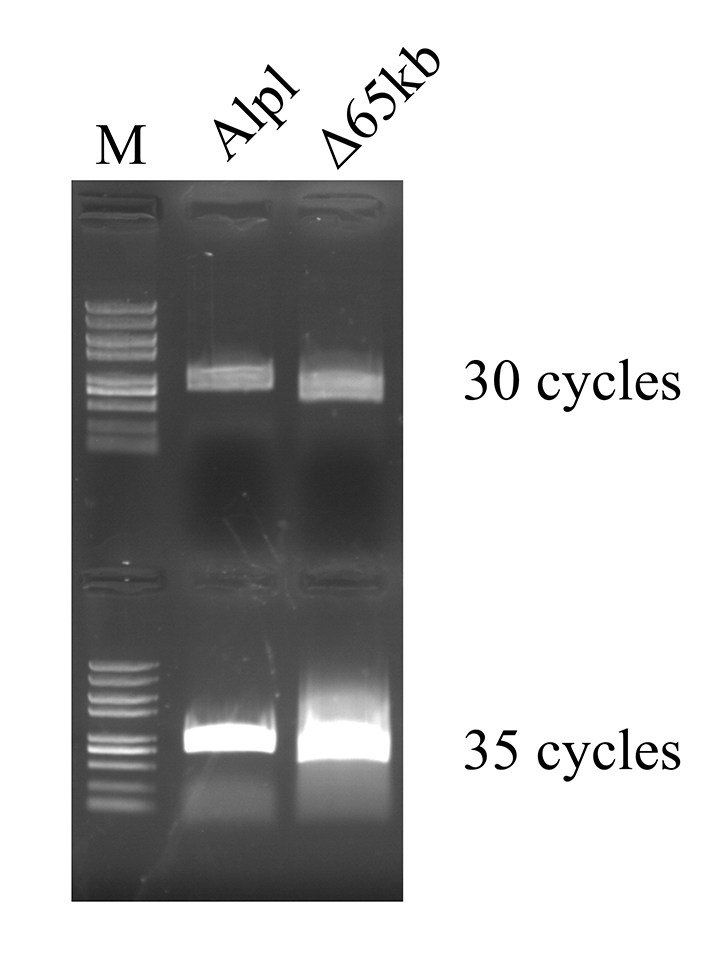

Supplement: S1 Fig — Genomic DNA from Dip2a Δ65kb/Δ65kb mice has been extracted and used to PCR for Alpl and Δ65kb locus for either 30 or 35 cycles. (TIF) [file pone.0120396.s001.tif]

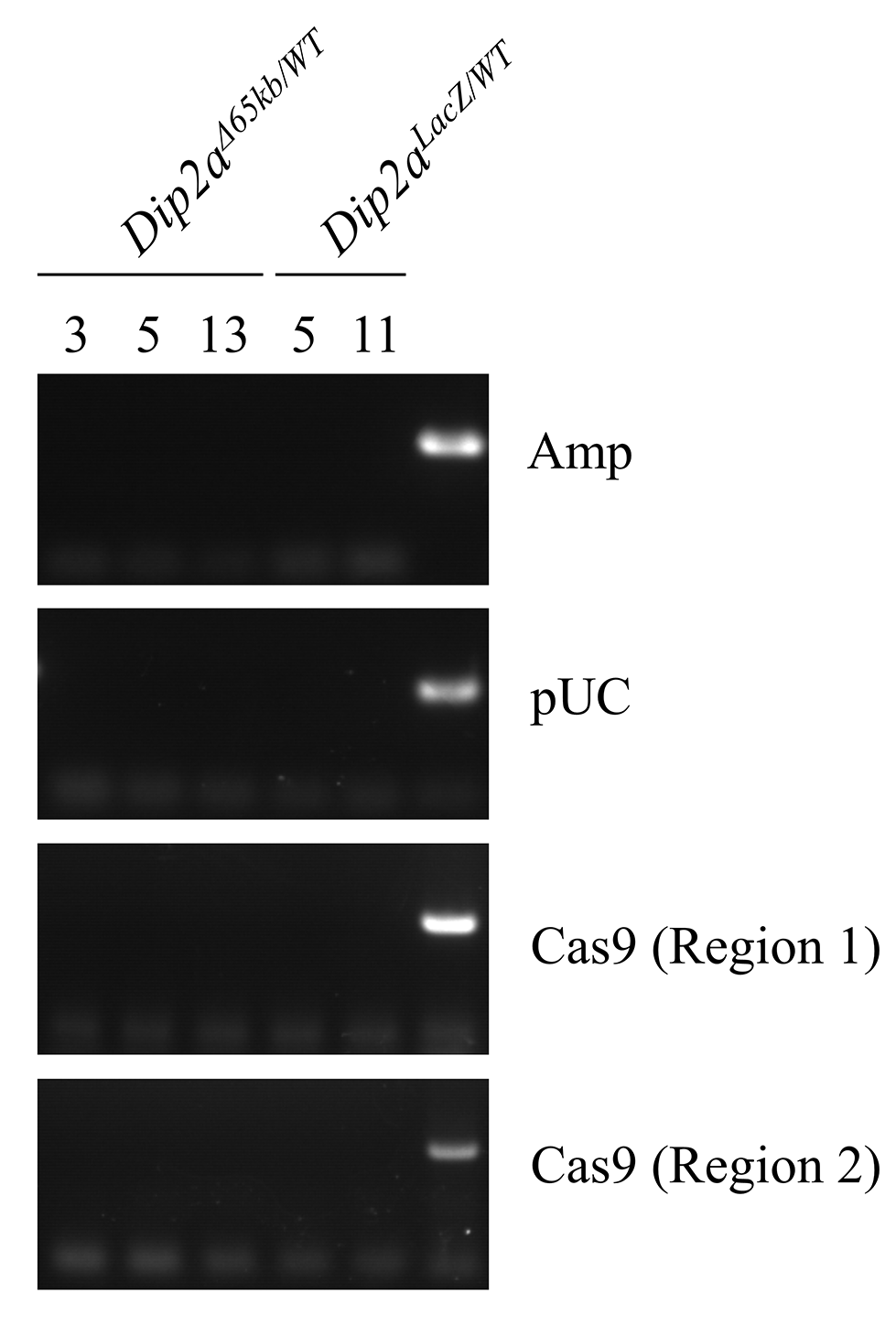

Supplement: S2 Fig — Genomic DNA from tail of Dip2a Δ65kb/WT and Dip2a LacZ/WT founder mice were subjected to PCR amplification for Ampicillin Resistant region (Amp), pUC replication origin (pUC), and two regions of Cas9 gene. Amp and pUC sequence exist on both DONOR and pX330 and Cas9 regions only exist on pX330 plasmid. (TIF) [file pone.0120396.s002.tif]

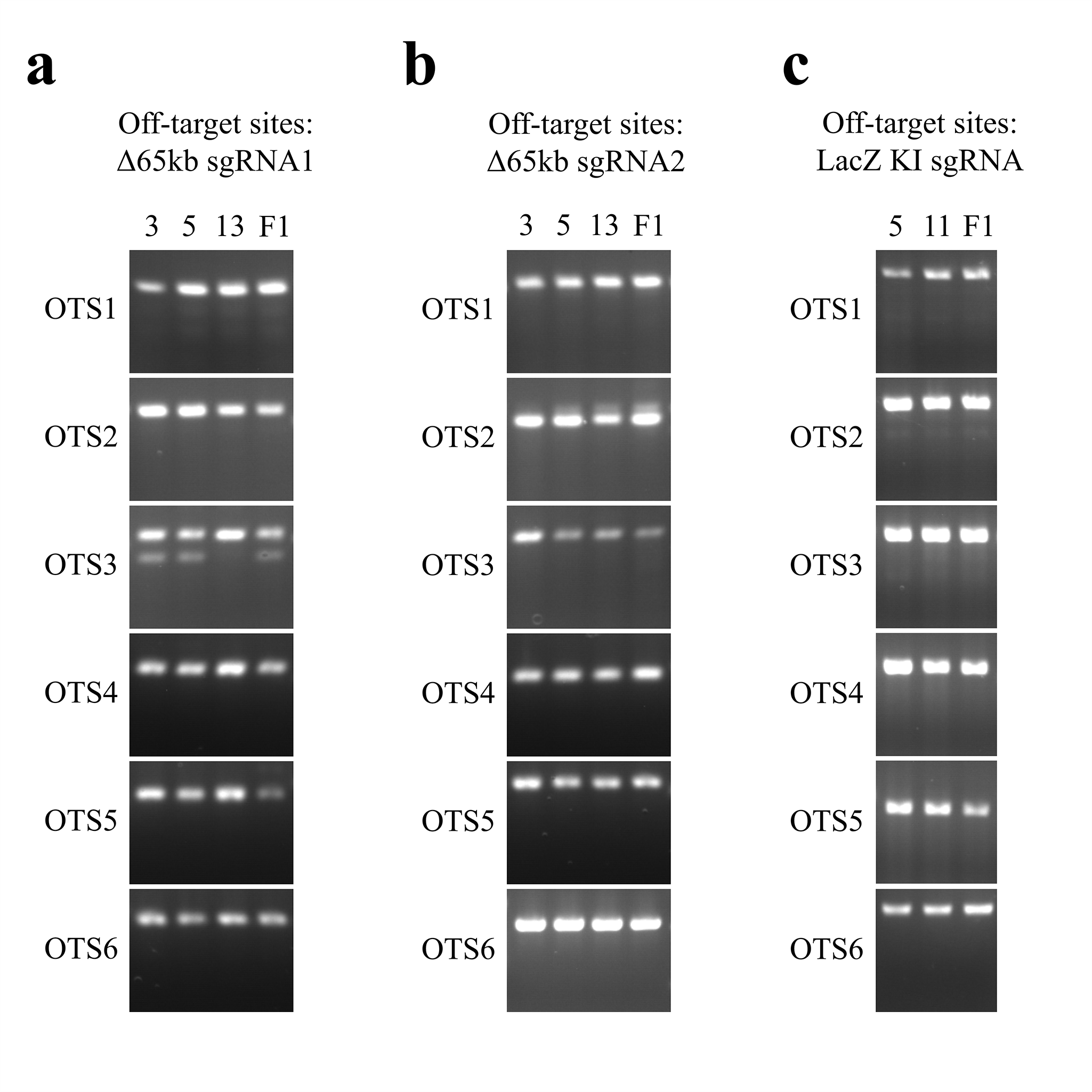

Supplement: S3 Fig — No obvious off target site observed. (TIF) [file pone.0120396.s003.tif]
