# Supplementary material for: Large Genomic Fragment Deletions and Insertions in Mouse Using CRISPR/Cas9
Source: PLoS One. 2015 Mar 24;10(3):e0120396. doi: 10.1371/journal.pone.0120396 (PMC4372442; doi:10.1371/journal.pone.0120396)
Supplement: S1 File — (DOCX) [file pone.0120396.s005.docx]

pL253-NEO-DTA

FRT-PGK-NEO-BGHpA-FRT-loxP-MC1-DTA-*TKpA*

tgcccgacggcgatgatctcgtcgtgacccatggcgatgcctgcttgccgaatatcatggtggaaaatggccgcttttctggattcatcgactgtggccggctgggtgtggcggaccgctatcaggacatagcgttggctacccgtgatattgctgaagagcttggcggcgaatgggctgaccgcttcctcgtgctttacggtatcgccgctcccgattcgcagcgcatcgccttctatcgccttcttgacgagttcttctgaggggatcaattctctagagctcgctgatcagcctcgactgtgccttctagttgccagccatctgttgtttgcccctcccccgtgccttccttgaccctggaaggtgccactcccactgtcctttcctaataaaatgaggaaattgcatcgcattgtctgagtaggtgtcattctattctggggggtggggtggggcaggacagcaagggggaggattgggaagacaatagcaggcatgctggggatgcggtgggctctatggcttctgaggcggaaagaaccagctggggctcgactagagcttgcggaacccttcgaagttcctattctctagaaagtataggaacttcatcagtcaggtacataatataacttcgtataatgtatgctatacgaagttattaggtggatctgatatccatatgctcgaggctagaactagtggatcctctagagtcgagcagtgtggttttcaagaggaagcaaaaagcctctccacccaggcctggaatgtttccacccaatgtcgagcagtgtggttttgcaagaggaagcaaaaagcctctccacccaggcctggaatgtttccacccaatgtcgagcaaaccccgcccagcgtcttgtcattggcgaattcgaacacgcagatgcagtcggggcggcgcggtcccaggtccacttcgcatattaaggtgacgcgtgtggcctcgaacaccgagcgaccctgcagcgacccgcttaacagcgtcaacagcgtgccgcagatcagccaccatggatcctgatgatgttgttgattcttctaaatcttttgtgatggaaaacttttcttcgtaccacgggactaaacctggttatgtagattccattcaaaaaggtatacaaaagccaaaatctggtacacaaggaaattatgacgatgattggaaagggttttatagtaccgacaataaatacgacgctgcgggatactctgtagataatgaaaacccgctctctggaaaagctggaggcgtggtcaaagtgacgtatccaggactgacgaaggttctcgcactaaaagtggataatgccgaaactattaagaaagagttaggtttaagtctcactgaaccgttgatggagcaagtcggaacggaagagtttatcaaaaggttcggtgatggtgcttcgcgtgtagtgctcagccttcccttcgctgaggggagttctagcgttgaatatattaataactgggaacaggcgaaagcgttaagcgtagaacttgagattaattttgaaacccgtggaaaacgtggccaagatgcgatgtatgagtatatggctcaagcctgtgcaggaaatcgtgtcaggcgatctctttgtgaaggaaccttacttctgtggtgtgacataattggacaaactacctacagagatttaaagctctaactcgac*aacacggaaggagacaataccggaaggaacccgcgctatgacggcaataaaaagacagaataaaacgcacgggtgttgggtcgtttgttcataaacgcggggttcggtcccagggctggcactctgtcgataccccaccgagaccccattggggccaatacgcccgcgtttcttccttttccccaccccaccccccaagttcgggtgaaggcccagggctcgcagccaacgtcggggcggcagg*ccctgccatagccacgggccccgtgggttagggacggggtcccccatggggaatggtttatggttcgtgggggttattattttgggcgttgcgtggggtcagtccacgactggactgagcagacagacccatggtttttggatggcctgggcatggaccgcatgtactggcgcgacacgaacaccgggcgtctgtggctgccaaacacccccgacccccaaaaaccaccgcgcggatttctggcgccgccggacgaactaaacctgactacggcatctctgccccttcttcgctggtacgaggagcgcttttgttttgtattggtcaccacgggtaccagcttttgttccctttagtgagggttaatttcgagcttggcgtaatcatggtcatagctgtttcctgtgtgaaattgttatccgctcacaattccacacaacatacgagccggaagcataaagtgtaaagcctggggtgcctaatgagtgagctaactcacattaattgcgttgcgctcactgcccgctttccagtcgggaaacctgtcgtgccagctgcattaatgaatcggccaacgcgcggggagaggcggtttgcgtattgggcgctcttccgcttcctcgctcactgactcgctgcgctcggtcgttcggctgcggcgagcggtatcagctcactcaaaggcggtaatacggttatccacagaatcaggggataacgcaggaaagaacatgtgagcaaaaggccagcaaaaggccaggaaccgtaaaaaggccgcgttgctggcgtttttccataggctccgcccccctgacgagcatcacaaaaatcgacgctcaagtcagaggtggcgaaacccgacaggactataaagataccaggcgtttccccctggaagctccctcgtgcgctctcctgttccgaccctgccgcttaccggatacctgtccgcctttctcccttcgggaagcgtggcgctttctcatagctcacgctgtaggtatctcagttcggtgtaggtcgttcgctccaagctgggctgtgtgcacgaaccccccgttcagcccgaccgctgcgccttatccggtaactatcgtcttgagtccaacccggtaagacacgacttatcgccactggcagcagccactggtaacaggattagcagagcgaggtatgtaggcggtgctacagagttcttgaagtggtggcctaactacggctacactagaagaacagtatttggtatctgcgctctgctgaagccagttaccttcggaaaaagagttggtagctcttgatccggcaaacaaaccaccgctggtagcggtggtttttttgtttgcaagcagcagattacgcgcagaaaaaaaggatctcaagaagatcctttgatcttttctacggggtctgacgctcagtggaacgaaaactcacgttaagggattttggtcatgagattatcaaaaaggatcttcacctagatccttttaaattaaaaatgaagttttaaatcaatctaaagtatatatgagtaaacttggtctgacagttaccaatgcttaatcagtgaggcacctatctcagcgatctgtctatttcgttcatccatagttgcctgactccccgtcgtgtagataactacgatacgggagggcttaccatctggccccagtgctgcaatgataccgcgagacccacgctcaccggctccagatttatcagcaataaaccagccagccggaagggccgagcgcagaagtggtcctgcaactttatccgcctccatccagtctattaattgttgccgggaagctagagtaagtagttcgccagttaatagtttgcgcaacgttgttgccattgctacaggcatcgtggtgtcacgctcgtcgtttggtatggcttcattcagctccggttcccaacgatcaaggcgagttacatgatcccccatgttgtgcaaaaaagcggttagctccttcggtcctccgatcgttgtcagaagtaagttggccgcagtgttatcactcatggttatggcagcactgcataattctcttactgtcatgccatccgtaagatgcttttctgtgactggtgagtactcaaccaagtcattctgagaatagtgtatgcggcgaccgagttgctcttgcccggcgtcaatacgggataataccgcgccacatagcagaactttaaaagtgctcatcattggaaaacgttcttcggggcgaaaactctcaaggatcttaccgctgttgagatccagttcgatgtaacccactcgtgcacccaactgatcttcagcatcttttactttcaccagcgtttctgggtgagcaaaaacaggaaggcaaaatgccgcaaaaaagggaataagggcgacacggaaatgttgaatactcatactcttcctttttcaatattattgaagcatttatcagggttattgtctcatgagcggatacatatttgaatgtatttagaaaaataaacaaataggggttccgcgcacatttccccgaaaagtgccacctctcaaggatcttaccgctgttgagatccagttcgatgtaacccattcgtgcacccaacttgatcttcagcatcttttactttcaccagcgtttctgggtgagcaaaaacaggaaggcaaaatgccgcaaaaaagggaataagggcgacacggaaatgttgaatactcatactcttcctttttcaatattattgaagcatttatcagggttattgtctcatgagcggatacatatttgaatgtatttagaaaaataaacaaataggggttccgcgcacatttccccgaaaagtgccacctgacgcgccctgtagcggcgcattaagcgcggcgggtgtggtggttacgcgcagcgtgaccgctacacttgccagcgccctagcgcccgctcctttcgctttcttcccttcctttctcgccacgttcgccggctttccccgtcaagctctaaatcgggggctccctttagggttccgatttagtgctttacggcacctcgaccccaaaaaacttgattagggtgatggttcacgtagtgggccatcgccctgatagacggtttttcgccctttgacgttggagtccacgttctttaatagtggactcttgttccaaactggaacaacactcaaccctatctcggtctattcttttgatttataagggattttgccgatttcggcctattggttaaaaaatgagctgatttaacaaaaatttaacgcgaattttaacaaaatattaacgcttacaatttccattcgccattcaggctgcgcaactgttgggaagggcgatcggtgcgggcctcttcgctattacgccagctggcgaaagggggatgtgctgcaaggcgattaagttgggtaacgccagggttttcccagtcacgacgttgtaaaacgacggccagtgaattgtaatacgactcactatagggcgaattggagctccaccgcggtggcggccggcgcgccgtcgacatcgattgcggccgccaattccgaagttcctattctctagaaagtataggaacttcaggtctgaagaggagtttacgtccagccaagctagcttggctgcaggtcgtcgaaattctaccgggtaggggaggcgcttttcccaaggcagtctggagcatgcgctttagcagccccgctgggcacttggcgctacacaagtggcctctggcctcgcacacattccacatccaccggtaggcgccaaccggctccgttctttggtggccccttcgcgccaccttctactcctcccctagtcaggaagttcccccccgccccgcagctcgcgtcgtgcaggacgtgacaaatggaagtagcacgtctcactagtctcgtgcagatggacagcaccgctgagcaatggaagcgggtaggcctttggggcagcggccaatagcagctttgctccttcgctttctgggctcagaggctgggaaggggtgggtccgggggcgggctcaggggcgggctcaggggcggggcgggcgcccgaaggtcctccggaggcccggcattctgcacgcttcaaaagcgcacgtctgccgcgctgttctcctcttcctcatctccgggcctttcgacctgcagcctgttgacaattaatcatcggcatagtatatcggcatagtataatacgacaaggtgaggaactaaaccatgggatcggccattgaacaagatggattgcacgcaggttctccggccgcttgggtggagaggctattcggctatgactgggcacaacagacaatcggctgctctgatgccgccgtgttccggctgtcagcgcaggggcgcccggttctttttgtcaagaccgacctgtccggtgccctgaatgaactgcaggacgaggcagcgcggctatcgtggctggccacgacgggcgttccttgcgcagctgtgctcgacgttgtcactgaagcgggaagggactggctgctattgggcgaagtgccggggcaggatctcctgtcatctcaccttgctcctgccgagaaagtatccatcatggctgatgcaatgcggcggctgcatacgcttgatccggctacctgcccattcgaccaccaagcgaaacatcgcatcgagcgagcacgtactcggatggaagccggtcttgtcgatcaggatgatctggacgaagagcatcaggggctcgcgccagccgaactgttcgccaggctcaaggcgcgca
