# Supplementary material for: Large Genomic Fragment Deletions and Insertions in Mouse Using CRISPR/Cas9
Source: PLoS One. 2015 Mar 24;10(3):e0120396. doi: 10.1371/journal.pone.0120396 (PMC4372442; doi:10.1371/journal.pone.0120396)
Supplement: S3 File — (DOCX) [file pone.0120396.s007.docx]

Dip2A delta65kb Donor vector

Dip2A5’Arm-NEO-Dip2A3’Arm

tcgacggataatgcaaattctggtcaagtgaaaatatatagacacaaagcagtacacacacatgtaacacacacatacacattcacttatacacatacacacatatacacacatagatgtacacacatacacacatatatacacatacctacacccacacacccacatacttttatatacatagatatacacacatacatgcatacacatacatacacacatatgcacacacatgtgcacatatacacacatacccacatacacccacatattcatatacacacacatacacacatggcaggagatgctctattatggacacactaaacgctacagaaagctgcttgtgaaggctagggcggaaccccgtgaggacggtgagatggggaaggatagcctgcctgcctccatggtctgttacccagcaggttctagacaaaagggcaggctagcggaaagggctctacgatagcccagctcacactatggcagctgcttgtctgtcccacagaccttcccctggctatggagtcatgtgcacccagaatgtttgtgaggcttaatctaagtgtcagacactccaaaggacctgttttcatacctacaaacctgagtcaaaatggcttcttctaaaggaactacttagctgtacactcaacagcagtattttctgatactctccagccttctactttttcatttggcagaaagtacaggaagaaggaggaggacttagaaaacaccaaaaatgcaaaaaagggaaaaaaaaggtggtagtgagaacggtgttaattaatacacatttgtactgaggcaactaataaaacctgttgcagtgctcgcttgtggtgtaagatcttcatgaaaaccttgaagagccactaccagcgctcccagttttgtgtagggacgtgaagaccaggaccaagtagaaaatgtgaagggcatttcctacgtcattgacaagtgggcggccgccaattccgaagttcctattctctagaaagtataggaacttcaggtctgaagaggagtttacgtccagccaagctagcttggctgcaggtcgtcgaaattctaccgggtaggggaggcgcttttcccaaggcagtctggagcatgcgctttagcagccccgctgggcacttggcgctacacaagtggcctctggcctcgcacacattccacatccaccggtaggcgccaaccggctccgttctttggtggccccttcgcgccaccttctactcctcccctagtcaggaagttcccccccgccccgcagctcgcgtcgtgcaggacgtgacaaatggaagtagcacgtctcactagtctcgtgcagatggacagcaccgctgagcaatggaagcgggtaggcctttggggcagcggccaatagcagctttgctccttcgctttctgggctcagaggctgggaaggggtgggtccgggggcgggctcaggggcgggctcaggggcggggcgggcgcccgaaggtcctccggaggcccggcattctgcacgcttcaaaagcgcacgtctgccgcgctgttctcctcttcctcatctccgggcctttcgacctgcagcctgttgacaattaatcatcggcatagtatatcggcatagtataatacgacaaggtgaggaactaaaccatgggatcggccattgaacaagatggattgcacgcaggttctccggccgcttgggtggagaggctattcggctatgactgggcacaacagacaatcggctgctctgatgccgccgtgttccggctgtcagcgcaggggcgcccggttctttttgtcaagaccgacctgtccggtgccctgaatgaactgcaggacgaggcagcgcggctatcgtggctggccacgacgggcgttccttgcgcagctgtgctcgacgttgtcactgaagcgggaagggactggctgctattgggcgaagtgccggggcaggatctcctgtcatctcaccttgctcctgccgagaaagtatccatcatggctgatgcaatgcggcggctgcatacgcttgatccggctacctgcccattcgaccaccaagcgaaacatcgcatcgagcgagcacgtactcggatggaagccggtcttgtcgatcaggatgatctggacgaagagcatcaggggctcgcgccagccgaactgttcgccaggctcaaggcgcgcatgcccgacggcgatgatctcgtcgtgacccatggcgatgcctgcttgccgaatatcatggtggaaaatggccgcttttctggattcatcgactgtggccggctgggtgtggcggaccgctatcaggacatagcgttggctacccgtgatattgctgaagagcttggcggcgaatgggctgaccgcttcctcgtgctttacggtatcgccgctcccgattcgcagcgcatcgccttctatcgccttcttgacgagttcttctgaggggatcaattctctagagctcgctgatcagcctcgactgtgccttctagttgccagccatctgttgtttgcccctcccccgtgccttccttgaccctggaaggtgccactcccactgtcctttcctaataaaatgaggaaattgcatcgcattgtctgagtaggtgtcattctattctggggggtggggtggggcaggacagcaagggggaggattgggaagacaatagcaggcatgctggggatgcggtgggctctatggcttctgaggcggaaagaaccagctggggctcgactagagcttgcggaacccttcgaagttcctattctctagaaagtataggaacttcatcagtcaggtacataatataacttcgtataatgtatgctatacgaagttattaggtggatctgatatcaaccggctaaagcagtgttttggatctgccaagtgtacctaccaaaacacaaggggaagaggcgggaagggaagggaaggggaggggaggggaggcctggtgttacactgtggctgttctccgctatactgcaaacttgcactttttttaggctaaaagaaattattcctgatttctaaatatatataatttcctgattatttattcccacttgtaataacaagcatatatgtaaggtctatgctggagagaacttttgagccaagcctgcccaggaaggcggatggaaagagccgccccattctccatgcccacagcccaggctccatgcagccgcacagcctcctttgtccagtgtgattactgaagcaggtgtgcctgctcgtctgtgtgtgtcgtcactaatgctcagcaagtgccattttggactgtttagttagatcctgtttcagaaagataaaccagcatttgatttaaaatcacttttgtagcctgtaacatagtgacgttaaactgcaagttctcatgagatcacagtattcatcctctcacgaagcacactgagttatttttatgagcagatctggacacatgagtggcagcctacagtcttctcattgtgctagccaggcctcttacacagctgagccgccatgggagtttgtctgtctagggtggacatagctgcagcctcttggctttgcatttgagttatgtgccctcgaggctagaactagtggatcctctagagtcgagcagtgtggttttcaagaggaagcaaaaagcctctccacccaggcctggaatgtttccacccaatgtcgagcagtgtggttttgcaagaggaagcaaaaagcctctccacccaggcctggaatgtttccacccaatgtcgagcaaaccccgcccagcgtcttgtcattggcgaattcgaacacgcagatgcagtcggggcggcgcggtcccaggtccacttcgcatattaaggtgacgcgtgtggcctcgaacaccgagcgaccctgcagcgacccgcttaacagcgtcaacagcgtgccgcagatcagccaccatggatcctgatgatgttgttgattcttctaaatcttttgtgatggaaaacttttcttcgtaccacgggactaaacctggttatgtagattccattcaaaaaggtatacaaaagccaaaatctggtacacaaggaaattatgacgatgattggaaagggttttatagtaccgacaataaatacgacgctgcgggatactctgtagataatgaaaacccgctctctggaaaagctggaggcgtggtcaaagtgacgtatccaggactgacgaaggttctcgcactaaaagtggataatgccgaaactattaagaaagagttaggtttaagtctcactgaaccgttgatggagcaagtcggaacggaagagtttatcaaaaggttcggtgatggtgcttcgcgtgtagtgctcagccttcccttcgctgaggggagttctagcgttgaatatattaataactgggaacaggcgaaagcgttaagcgtagaacttgagattaattttgaaacccgtggaaaacgtggccaagatgcgatgtatgagtatatggctcaagcctgtgcaggaaatcgtgtcaggcgatctctttgtgaaggaaccttacttctgtggtgtgacataattggacaaactacctacagagatttaaagctctaactcgacaacacggaaggagacaataccggaaggaacccgcgctatgacggcaataaaaagacagaataaaacgcacgggtgttgggtcgtttgttcataaacgcggggttcggtcccagggctggcactctgtcgataccccaccgagaccccattggggccaatacgcccgcgtttcttccttttccccaccccaccccccaagttcgggtgaaggcccagggctcgcagccaacgtcggggcggcaggccctgccatagccacgggccccgtgggttagggacggggtcccccatggggaatggtttatggttcgtgggggttattattttgggcgttgcgtggggtcagtccacgactggactgagcagacagacccatggtttttggatggcctgggcatggaccgcatgtactggcgcgacacgaacaccgggcgtctgtggctgccaaacacccccgacccccaaaaaccaccgcgcggatttctggcgccgccggacgaactaaacctgactacggcatctctgccccttcttcgctggtacgaggagcgcttttgttttgtattggtcaccacgggtaccagcttttgttccctttagtgagggttaatttcgagcttggcgtaatcatggtcatagctgtttcctgtgtgaaattgttatccgctcacaattccacacaacatacgagccggaagcataaagtgtaaagcctggggtgcctaatgagtgagctaactcacattaattgcgttgcgctcactgcccgctttccagtcgggaaacctgtcgtgccagctgcattaatgaatcggccaacgcgcggggagaggcggtttgcgtattgggcgctcttccgcttcctcgctcactgactcgctgcgctcggtcgttcggctgcggcgagcggtatcagctcactcaaaggcggtaatacggttatccacagaatcaggggataacgcaggaaagaacatgtgagcaaaaggccagcaaaaggccaggaaccgtaaaaaggccgcgttgctggcgtttttccataggctccgcccccctgacgagcatcacaaaaatcgacgctcaagtcagaggtggcgaaacccgacaggactataaagataccaggcgtttccccctggaagctccctcgtgcgctctcctgttccgaccctgccgcttaccggatacctgtccgcctttctcccttcgggaagcgtggcgctttctcatagctcacgctgtaggtatctcagttcggtgtaggtcgttcgctccaagctgggctgtgtgcacgaaccccccgttcagcccgaccgctgcgccttatccggtaactatcgtcttgagtccaacccggtaagacacgacttatcgccactggcagcagccactggtaacaggattagcagagcgaggtatgtaggcggtgctacagagttcttgaagtggtggcctaactacggctacactagaagaacagtatttggtatctgcgctctgctgaagccagttaccttcggaaaaagagttggtagctcttgatccggcaaacaaaccaccgctggtagcggtggtttttttgtttgcaagcagcagattacgcgcagaaaaaaaggatctcaagaagatcctttgatcttttctacggggtctgacgctcagtggaacgaaaactcacgttaagggattttggtcatgagattatcaaaaaggatcttcacctagatccttttaaattaaaaatgaagttttaaatcaatctaaagtatatatgagtaaacttggtctgacagttaccaatgcttaatcagtgaggcacctatctcagcgatctgtctatttcgttcatccatagttgcctgactccccgtcgtgtagataactacgatacgggagggcttaccatctggccccagtgctgcaatgataccgcgagacccacgctcaccggctccagatttatcagcaataaaccagccagccggaagggccgagcgcagaagtggtcctgcaactttatccgcctccatccagtctattaattgttgccgggaagctagagtaagtagttcgccagttaatagtttgcgcaacgttgttgccattgctacaggcatcgtggtgtcacgctcgtcgtttggtatggcttcattcagctccggttcccaacgatcaaggcgagttacatgatcccccatgttgtgcaaaaaagcggttagctccttcggtcctccgatcgttgtcagaagtaagttggccgcagtgttatcactcatggttatggcagcactgcataattctcttactgtcatgccatccgtaagatgcttttctgtgactggtgagtactcaaccaagtcattctgagaatagtgtatgcggcgaccgagttgctcttgcccggcgtcaatacgggataataccgcgccacatagcagaactttaaaagtgctcatcattggaaaacgttcttcggggcgaaaactctcaaggatcttaccgctgttgagatccagttcgatgtaacccactcgtgcacccaactgatcttcagcatcttttactttcaccagcgtttctgggtgagcaaaaacaggaaggcaaaatgccgcaaaaaagggaataagggcgacacggaaatgttgaatactcatactcttcctttttcaatattattgaagcatttatcagggttattgtctcatgagcggatacatatttgaatgtatttagaaaaataaacaaataggggttccgcgcacatttccccgaaaagtgccacctctcaaggatcttaccgctgttgagatccagttcgatgtaacccattcgtgcacccaacttgatcttcagcatcttttactttcaccagcgtttctgggtgagcaaaaacaggaaggcaaaatgccgcaaaaaagggaataagggcgacacggaaatgttgaatactcatactcttcctttttcaatattattgaagcatttatcagggttattgtctcatgagcggatacatatttgaatgtatttagaaaaataaacaaataggggttccgcgcacatttccccgaaaagtgccacctgacgcgccctgtagcggcgcattaagcgcggcgggtgtggtggttacgcgcagcgtgaccgctacacttgccagcgccctagcgcccgctcctttcgctttcttcccttcctttctcgccacgttcgccggctttccccgtcaagctctaaatcgggggctccctttagggttccgatttagtgctttacggcacctcgaccccaaaaaacttgattagggtgatggttcacgtagtgggccatcgccctgatagacggtttttcgccctttgacgttggagtccacgttctttaatagtggactcttgttccaaactggaacaacactcaaccctatctcggtctattcttttgatttataagggattttgccgatttcggcctattggttaaaaaatgagctgatttaacaaaaatttaacgcgaattttaacaaaatattaacgcttacaatttccattcgccattcaggctgcgcaactgttgggaagggcgatcggtgcgggcctcttcgctattacgccagctggcgaaagggggatgtgctgcaaggcgattaagttgggtaacgccagggttttcccagtcacgacgttgtaaaacgacggccagtgaattgtaatacgactcactatagggcgaattggagctccaccgcggtggcggccggcgcgccg
