# Supplementary material for: Large Genomic Fragment Deletions and Insertions in Mouse Using CRISPR/Cas9
Source: PLoS One. 2015 Mar 24;10(3):e0120396. doi: 10.1371/journal.pone.0120396 (PMC4372442; doi:10.1371/journal.pone.0120396)
Supplement: S4 File — (DOCX) [file pone.0120396.s008.docx]

pTie2-LacZ-YWZ

Tie2-promoter-sequence-IRES-LacZ-Tie2-Enhancer-sequence

ggcggccgcgagctcaattaaccctcactaaagggagtcgactcgatccgctctctgcctgtaccaccctctggcatttaatcacacaatgcttggttttgttcttcaacttttcctgttatgatgcagtccctggcttgtgtaactatgagcttcaaaagcaaagaacgcatcatctatttttgtgtctcttcttccaaggacttagtgtatcacttactggctaaatgcttgagacaaaaacagggattaatgaagaagaaagagaaagaaaaggaagggaaagtgcccacaattactgacagggtttcagtaaagcagtctagagggtcaggtattttccatagccatgccccagagtgggtgttgccactttagctgccctggtctggctgaaggccaggacttgattgttgatggcccttcctttgctgctagtcactgttaagtactgcagatttacagaaagcttcatggaggtctgtaagaagccagaggtgataacaccaagatttagagccactgaccagcagaatgcagaatgtccaggctatgatccaggttgtagatcctgatctgactactcaagactggttgaaggcaaggttcacttggattcactctatttgccagcagatgttttaaatccatcatatatatatatatatctccattactttaggacagtggttctcagccttcctaatgctgtagccctttaatagagttcctcatattgtgattgtaaaaattattttgttgctacttcatgactaattttgctactgtgaaagggtcattttaccccaggctgttgagaccacatgttgggaaccactactttagaaggcattggggttggagaagaacatgaagaatagagtaacagtggtcagttttggttcattatatcacagaaacattcactttaaggtttcagcatgtttgttgtgtatatgtgattgtgtaaagacttcaccaggtctttctttaatcaccatacctaacatcttcaccactccatatccatcagcttcaccttgtactctagcatttgggcattcatcctgtaccagggcaggcatccattcttttgcaactcacattgtttcctagttttgattattaccaacaatgcttctagaccatgaattttggtctttgacttttgcttggtaaacatcataaaacaatccagtggtggtggtggtgccgctgctgctggtggtggtggtaaagcaggaagccataaagtgcctttattcaatctgtatttgatacaaattgttatttcttcccatgtaaaagatatggcatctgaagtgtagaggtctgaattcaaacctccacatcaccagatagtatattacagactcaacaaataatacacggctttgcctgacttcaaagccctgttcttgacgtaagtatatgagtaacaatggtagcaccttagtttttatcagttcactaaatatttatataagacctactatgaagggagatagaagggtatgaggtggggtcatgggaataggaaaacggtggaagggagaaggagaattaacaaaagctaattatgtttgaaaatgccacaatgaaacctaatttacaaaagaaccactatatgaccttcacagtgtgtgctaagtcttggagatttagtggtgaagaagtcaggtgtgtttccaatctcatggaggatgtaatcagttagagagcacaggagcacataaaaagataggcaaaaatgtatgattagtaccatgtaagatatgaaggggaacacaggaaactagtggggagacctaatttagtttgagtggtcttcaaagaccctttagaagctgagaactaaagacagcaagcaaggtgagggcagcatctccacctttccagtggaatgagcaacttaagggtataacagctgattcccacattgtcaacaaggctcttcagagactagagatgcactaatgatgaccatacccagcttttaaggaaggtttctgagcatgtccaagcaccctacactaggcattggaaatcaacatgtccagagatggaagtgacagtcagtaagccaacccttttcaaaacttccaaagctattactcgtcaactctccagacatatgggccccgagtgtgttgggaagctctcattattgttctttgattggttctctacattccgagatccaaggagcagttatctcaggtagaggatcgtggaatgtctgcccatgattaacttcaatttatacctgtaagttataccacatcctaaacacgctgatgtcccagagaacattttgaccagctgctaacaaaacccaggagcatttagaaaaaaactgagtcacccaccgttctggataatgatggagagaaacaaatgggattattcttacagagtatgaaagttacataattttcctggataatggagaattaattaaacatcagcatcttttctggactgcagagggaagacagaggtgaagccaatctttccgggaaatggaggaggaaagaatttgactactatttgggggttaacaatacatcttactagcatggcaaaggaaactgggctgcttttcagagtaagccaccccagtagatgctgcaaggctgtgctttcatcccaggagaaagtcaacagggccaggcatgccagaacatgcccataatgtaaccacttaggctgaggcagaaagatcaaaaatcccaggccagcttagtttgtgtaacaagacctttgctcaaacaaagatttacaaaacaaacaagcaaacaaacaaatataaaaaaggagaagaaaataactgccaggggaggctgtgagcaatgaagacttgatgagtgaccatctcgcacagtggacgcttgtgtctagaaggtaagggcttggcaatgtttcccaggttttccattcctggtttatatggcttgaggccagtggacttcacaatgtctcagcttccaggtctttatacagagcatattagccacatgtggtagcttgtgcctgtaatgctggcacttgagagaccaagacaggaggattgccacaagtctccatccagcctaggtgctgtgtcactctgtctcacccctgacccagtcccacccaacatcaaacaggctatcactgtgacactggtactgagtcagaatcacccagattaaagattctgggagatcagtcctggggatgcgggaagtgagaccagttatttaataattcttatactcatgagatgatggatccagatgagaaattgtaaaaattttaggttttataattgaagaaataggtggtttcttcaggttacatctctccactgttggtcatttcagctaaggtcactccccattgattcctgtgaggctctcacatcccaggtctctgggactttctagaggttcccgctgcttcccagccctgaaaatgcgtatttctattcattctcctggcattctgggcttctctcctgtcccccgccccacccaacacctgatcctgccccctttctctcccccttctctctctaaaccaggtccctccctccctctgcttcccatgattattttgttccctcctctaaatgagtctgaagcatcctcacttggaccttccttcttgttaaacttcatatggtctgtgagttgtatcatgggtattctgtacttttttggctaatgtttcacttatcagtgagtgcaaaccaggcatatccttttgagtttgggttacctcactcaggatgatattttctagttctatccattcgcctgcaaaattcatgatgtcctaatttttagtagctgaatagtattccattgtgtaaatgaaccatattttctgcatctgttcttcagctgagggaaatctgggttgtttccagcttctaggtattataaataaggttgctatgaacatagtggaacacatatccttgaggtatggtagagcatcttttgggtatatatccaggagtggatagttgggttttcaggtagaactatttccaattttctaaggaaccaccagattgatttttagatagacagggcccctagtggagagatggggccaaacacctaccttcaaaaatttggtccagaattgttcctctctaaaagaaatgcaggaaattcatgatgtcctaatttttagtagctgaatagtattccattgtgtaaatgaaccatattttctgcatctgttcttcagctgagggaaatctgggttgtttccagcttctaggtattataaataaggttgctatgaacatagtggaacacatatccttgaggtatggtagagcatcttttgggtatatatccaggagtggatagttgggttttcaggtagaactatttccaattttctaaggaaccaccagattgatttttagatagacagggcccctagtggagagatggggccaaacacctaccttcaaaaatttggtccagaattgttcctctctaaaagaaatgcagggacaaaaatgaaacagagactgaccaacccaacttaggatccatcctatgggcaagcaccaaacccagactctattattgatgccatgttgtgcttgcagacaggagcttagcatggctgtcctctgagacactctatcagcagctgactgggacagatgcagatgccaacccttgaactgaggtccaggacccctatggaagaattaggggaaggtttgaaggagctgaaggggatggcaaccccataggaaaaacaagtgtcaactaaccctcagagctcccagagactaagccaccaactaaagagcatacatgggctggtttgtggtccctggcagaggactgccttgtctggcctcagtaggagaggatgtgcctaatcctctagagacttgatgccccagggaaggggacaaggaggggacaaggtggagattggtgtggggtagtgggggttgggggtgggggtggggatgtgaatgggtgagtgagggagggaatgagtgagtgggtggtacagcatcctctcagaggcaaaggggaaggggagtggataacaaactctgggagcagggacggggaaggagggcaacatttgtaattaaataaataaaataatttaataaaaaaaatgaagaaacaggataacttgggaatggttacagcagggctgggattagaacccaaaaagtttattctgagactcttttccaataccaagcttaaagttttcttcagaattctatagaatgcctttttggcagaagttctttggactttaataaagaacatattgaagagatgaaaagaagcttactaagatctaatgaaaatcaagatgttaggcacagtgccagatactttaacatagtaatatgactctttagagttttgagacagggcctcatatagtttatgatgaattcactgttttgtcaaagatgaccttgaactcttaatccattcccaaagtgttgttgtcatatgtttgcaccactcctggcttcatagtgtttttaaaacacccatggagagtcgggtgtgaagatccacacgtctaacctcagcatctggtgaatcaaggcaggagggcgggtggttgcaggctggctataatatctaagtttcagttagtaagggctgcataatgaaacactgtcttaaacacaaaaccaaaacccatgaaggagatactattgccatttaaaagtctctggaatggaaatagctatcataatcttacctctgagccagtgtctgccctcaggtgtgcctgaggactgaacagggctatgcactcctcaggttggaaacattactagtcctcagtgtctgctcttgacctgttaacagctgagtcagggtctgccctcagctgtgcctgaggacagagctgagctatctacccctgcagattggaagcattacaggcactcaagatcagccctgaagtgataaaacctaaggcagaaatccaccaagactagcagtgcctccgtgtctcttcctgtggctggtgggaaagggaggggcagtccttccttgatgcaaggtcgtgtgtctagtggcatgcttgcttcattcccagtgagagcaagtgatcacctgggtaaggaaggttcaggtgcctgagcttgctggagaattcatcactcatccatcactctgctcctgtagacataatcacttctgttgggtctttatagagatgatttataactttgttgtttatagtttttatgaatgtgtgtattcatttaggtcacatgggaggtgcacattttcaggtgtctgtctttccatcacacgggctttgaattaaactcagtcttggttttaccggctgagccatctcacctgcctgattatttaaaaatctccggagtaatccaagagtgtggtttatgattgtagtatcaacactcgggaggctgagggagcatcgttatcatgagctccaggctagttccaggcttgcctaagctgtagagcaagtcactctcttaaaaagtgcctctcccatatttttgtatataatttgcatctgaaattctgtttgccaataactatgaaattattcacattactaaaatcttcctgtgccaagttctccaacgaattagatcacactcagatgaaatgctaataaaaattaaagctgtagccagtagcatgcgtatatttgggctcagggccaacaggcaggcgatctgggtgtaagaaaataggctaatggctgtggaatctggtctctagtggctccgctgagagctgacctcaaccacgctccctcaaattgattgccttccaggttatgatttctcatcacaggaaactttgttgcccaattcaaaccctgtgagtgaaaacaaaaacaggagagcaagtgctgctccccgtgccccaaagccccttctgtcagggatcccaaatgcaccccagagaacagcttagcctgcaagggctggtcctcatcgcataccatacataggtggagggcttgttattcaattcctggcctatgagaggatacccctattgttcctgaaaatgctgaccaggaccttacttgtaacaaagatccctctgccccacaatccagttaaggcaggagcaggagccggagcaggagcagaagataagccttggatgaagggcaagatggatagggctcgctctgccccaagccctgctgataccaagtgcctttaagatacagcctttcccatcctaatctgcaaaggaaacaggaaaaaggaacttaaccctccctgtgctcagacagaaatgagactgttaccgcctgcttctgtggtgtttctccttgccgccaacttgtaaacaagagcgagtggaccatgcgagcgggaagtcgcaaagttgtgagttgttgaaagctcacgtgggcgcgctagcatttaaattcgaaggcgcgccttaattaagaattccgccccccccccccccccctctccctcccccccccctaacgttactggccgaagccgcttggaataaggccggtgtgcgtttgtctatatgttattttccaccatattgccgtcttttggcaatgtgagggcccggaaacctggccctgtcttcttgacgagcattcctaggggtctttcccctctcgccaaaggaatgcaaggtctgttgaatgtcgtgaaggaagcagttcctctggaagcttcttgaagacaaacaacgtctgtagcgaccctttgcaggcagcggaaccccccacctggcgacaggtgcctctgcggccaaaagccacgtgtataagatacacctgcaaaggcggcacaaccccagtgccacgttgtgagttggatagttgtggaaagagtcaaatggctctcctcaagcgtattcaacaaggggctgaaggatgcccagaaggtaccccattgtatgggatctgatctggggcctcggtgcacatgctttacatgtgtttagtcgaggttaaaaaaacgtctaggccccccgaaccacggggacgtggttttcctttgaaaaacacgatgataatatggccacaaccatggctccaaaaaagaagagaaaggtagaagacccccgcatggctcgcgatgatcccgtcgttttacaacgtcgtgactgggaaaaccctggcgttacccaacttaatcgccttgcagcacatccccctttcgccagctggcgtaatagcgaagaggcccgcaccgatcgcccttcccaacagttgcgcagcctgaatggcgaatggcgctttgcctggtttccggcaccagaagcggtgccggaaagctggctggagtgcgatcttcctgaggccgatactgtcgtcgtcccctcaaactggcagatgcacggttacgatgcgcccatctacaccaacgtgacctatcccattacggtcaatccgccgtttgttcccacggagaatccgacgggttgttactcgctcacatttaatgttgatgaaagctggctacaggaaggccagacgcgaattatttttgatggcgttaactcggcgtttcatctgtggtgcaacgggcgctgggtcggttacggccaggacagtcgtttgccgtctgaatttgacctgagcgcatttttacgcgccggagaaaaccgcctcgcggtgatggtgctgcgttggagtgacggcagttatctggaagatcaggatatgtggcggatgagcggcattttccgtgacgtctcgttgctgcataaaccgactacacaaatcagcgatttccatgttgccactcgctttaatgatgatttcagccgcgctgtactggaggctgaagttcagatgtgcggcgagttgcgtgactacctacgggtaacagtttctttatggcagggtgaaacgcaggtcgccagcggcaccgcgcctttcggcggtgaaattatcgatgagcgtggtggttatgccgatcgcgtcacactacgtctgaacgtcgaaaacccgaaactgtggagcgccgaaatcccgaatctctatcgtgcggtggttgaactgcacaccgccgacggcacgctgattgaagcagaagcctgcgatgtcggtttccgcgaggtgcggattgaaaatggtctgctgctgctgaacggcaagccgttgctgattcgaggcgttaaccgtcacgagcatcatcctctgcatggtcaggtcatggatgagcagacgatggtgcaggatatcctgctgatgaagcagaacaactttaacgccgtgcgctgttcgcattatccgaaccatccgctgtggtacacgctgtgcgaccgctacggcctgtatgtggtggatgaagccaatattgaaacccacggcatggtgccaatgaatcgtctgaccgatgatccgcgctggctaccggcgatgagcgaacgcgtaacgcgaatggtgcagcgcgatcgtaatcacccgagtgtgatcatctggtcgctggggaatgaatcaggccacggcgctaatcacgacgcgctgtatcgctggatcaaatctgtcgatccttcccgcccggtgcagtatgaaggcggcggagccgacaccacggccaccgatattatttgcccgatgtacgcgcgcgtggatgaagaccagcccttcccggctgtgccgaaatggtccatcaaaaaatggctttcgctacctggagagacgcgcccgctgatcctttgcgaatacgcccacgcgatgggtaacagtcttggcggtttcgctaaatactggcaggcgtttcgtcagtatccccgtttacagggcggcttcgtctgggactgggtggatcagtcgctgattaaatatgatgaaaacggcaacccgtggtcggcttacggcggtgattttggcgatacgccgaacgatcgccagttctgtatgaacggtctggtctttgccgaccgcacgccgcatccagcgctgacggaagcaaaacaccagcagcagtttttccagttccgtttatccgggcaaaccatcgaagtgaccagcgaatacctgttccgtcatagcgataacgagctcctgcactggatggtggcgctggatggtaagccgctggcaagcggtgaagtgcctctggatgtcgctccacaaggtaaacagttgattgaactgcctgaactaccgcagccggagagcgccgggcaactctggctcacagtacgcgtagtgcaaccgaacgcgaccgcatggtcagaagccgggcacatcagcgcctggcagcagtggcgtctggcggaaaacctcagtgtgacgctccccgccgcgtcccacgccatcccgcatctgaccaccagcgaaatggatttttgcatcgagctgggtaataagcgttggcaatttaaccgccagtcaggctttctttcacagatgtggattggcgataaaaaacaactgctgacgccgctgcgcgatcagttcacccgtgcaccgctggataacgacattggcgtaagtgaagcgacccgcattgaccctaacgcctgggtcgaacgctggaaggcggcgggccattaccaggccgaagcagcgttgttgcagtgcacggcagatacacttgctgatgcggtgctgattacgaccgctcacgcgtggcagcatcaggggaaaaccttatttatcagccggaaaacctaccggattgatggtagtggtcaaatggcgattaccgttgatgttgaagtggcgagcgatacaccgcatccggcgcggattggcctgaactgccagctggcgcaggtagcagagcgggtaaactggctcggattagggccgcaagaaaactatcccgaccgccttactgccgcctgttttgaccgctgggatctgccattgtcagacatgtataccccgtacgtcttcccgagcgaaaacggtctgcgctgcgggacgcgcgaattgaattatggcccacaccagtggcgcggcgacttccagttcaacatcagccgctacagtcaacagcaactgatggaaaccagccatcgccatctgctgcacgcggaagaaggcacatggctgaatatcgacggtttccatatggggattggtggcgacgactcctggagcccgtcagtatcggcggaattccagctgagcgccggtcgctaccattaccagttggtctggtgtcaaaaataataataaccgggcaggggggatctaagctctagataagtaatgatcataatcagccatatcacatctgtagaggttttacttgctttaaaaaacctcccacacctccccctgaacctgaaacataaaatgaatgcaattgttgttgttaacttgtttattgcagcttataatggttacaaataaagcaatagcatcacaaatttcacaaataaagcatttttttcactgcattctagttgtggtttgtccaaactcatcaatgtatcttatcatgtctggatcaaatggcgccagcttcccagggactcatgctcatctgtggacgctggatggggagatctggggaagtatggactctttagccggcttagttctctgtggagtcagcttgctcctttatggtaagttttggcttgatgtttatttgtgtgtgtgtgtcatgttttaacaacagtgacttctcgccattctctctctcaccaaaccttcgatttggtgaccctgacactgcttttctgagactctccagtttacacatggcaacggttttgaagttcagattccagcggcaccagctgggttttctagccatctcttgtagacagatgctgcctcctgggttgccacggtctctcccaggactgagtaagtcaccttaattgaagggatctgtttttttttttttttttttttttttggagtattctttcttgcctctcattctggtgttttcttatccataacaacaaatttgggttgtaatcactttaaagaaagacttcaatgatttttttttttttttttggtagaaacaaaattcttacctagccatgtacaaaaacgtcattgttaactaaaaacaagaccacttttctgtaaagattgtgtatctccatgctttttaatttcagtagtccagttaagggcttttcgtcttttaggtaggggtccaggagttagggctatgccactgactttaattttttgatgtttcacataaagattagccctagcaagctaactaaaatgtagtcaattaccaagctcaccggattcagtattttgccaagtcctgtagtggcacctctagagagggcagccaggatgagcttgggttctgtatgtgtatcacaggctctacacagtggggtgccttgtaactcactcagcataggtaaaccatgctaggagaaacatggggacccaaactgcagatcacagacacattatcattgccataggattccccgaattggtttggaagtattctaattattatgataaaaatgtaattgacacaggagatgaatttgggaaaatttatcaaacttaatggtgaggctatatatagtatagcatttgatacaaagaaatacttttgatgttacttttatatttttctatatgtttaaatattttgttaagtattcaaaaaaatttaacccagtaaagtatgataattgtgtgcaatttctcatgtaacttttcaatttgatttcaacaaccaacatatcagaaatttaataatatttaagagtaatgtaaattgctgaaagagtatctaagatggtgccaattacttatgtagtaacttacttataattaacataacagcataatatattgctgtaaatttggattcgtctttatattaatgtagagacaggttgatgtctttgttactatgtacttttaaataataattatggttcctcactttcatcttgctttccacaagtcacactgatcagcgttatagaatatgggactgattttacaccagagactataataatacaaagtccaagtatctagttctgctctgacttagttttaagttaaataaactaatagtatgttcacatacgtatgtgcacgcacacatacacagaactattttaacttagtctttctccttaagtgacttttttatggcattttacaaataagtttagaaaagttgtcatgaattcatacaaaaaagttctgttccaaaacagttgtctcactgatgctagaaactgatttctggagcgaagatgacaagaaggaggaggtcatgttcttgtcagaaagtagaacacagaaagcttggtttctgctgaaggtctctgttaattcctgactttcattctgtacccaagcttccatgaacttgagtgaggcttggtgatcacacaaacagcctcaggagttcttccttcaagtcagactctaatagtgtacttcattaatgtggaaggggatatagagcttatatgtctagtctacttggtggcctgatattccttttttgttgaacaaacataatggaagaataaagactctgtatttgcctcaataaaggttgctctgggtagcaaagcagcctgctaaggcataaactggaagaggtttgtggcttccataacaactcagattctggcgcttgctccttccactggagtattattgccaatttcagcttgcaaacaagttctcatagaacttagcagtgttaccccctctttaattccatatctacaccaaacctggctgcaggctctaaagaattgtgtacacttaaaaaaaaaaaaaaaacaccacttctgtttcatcatggatgtcctatatgattcacactgataggaagtcttcaggtcacacatcttcagctgtctttcctgtcttgtgtgacattttaaacaactggattcagagataacagagatgttgtctgggctggcaaaggaaacgcaatagtaaggtcaggaaagtggtcccaggagccttctccttggactgggatcaggacacctcttgggattgttttccaagggaagatctggccttctacagaaactctcccttccaaccattgctcttgttagcacttcccccttagccctaccctgaaagaacttccaggaagagtctaatttttctatgacatctctccaggaggaaagaaattatccaagtcgatctcaaaagactatgctgtgtccttttagaactttcctaatgctgtgaccctttaatacagttcttcatggtgtggtgagccccagccataaaattattttcattgctacttcataactataattttgctactgttgtgaatcataatgtaaatatctgataggtgaccctcaaaagggtcatgattcacaggttgagaatcacttccctaggggctctgaattcttccaaagatatcagtttgttacttacagttccaccgatgtcaaaagacaggtgtgaccaagttacctctgacccctagtagaaggttttctagttagcacaccaaatcaggattcaggaagtttctttaatctggaaagatgggtccaaagcaaaagacatcattttaaaaatagtgaaatgtcaactttgttatttgggttaaaaaaatagattatggatactcattttaggagaaaagtggttagtttaaaattcttgtggataataagtagtttttgatgagacacaaaattaataggagctctgcttttatgaaatatgatgcataaattaggtgcaagtaagaaatgatatcacttcaacatgctgcttagagtacacccattaaacattgcagagaaccttcacagcaaactagcaggcaagatgcaacagtttcagacatggatgaggaatcacaaattggagtttacaacattgaagcccaaagtggataagcacagcacattcatctttcatcttttgggatgagaatattcagttcttagggttgctctaaggatcaaagatgctgctgcatgtcaaatgttagtcagtaagaactccctcagtcagtataactaaaggagtcatctgctaccagcatctgtacaaagcagcagtgacactatctttcccagcatctaggtaaagctctcagtacttgtagtctttttccacaatatctcaacatggttgagcccttcaaggttttatctgttcatccctggtcatggtacatatcaaggccacatcaaaatctatacatccttttgtctcagcccttgttcatggaatcttacccttttggaaacagcaaattaaaaaatgtaaaagctcattttatttgtgtgggtgttttggctgcatctgtctatgtaccatgtgtgtgcccgatgccaggggagaccagaagaggttactggatccctgggacttacagagttatagatagttgtaagctgctatatagatgttgggaatccaacctaggtccttaggaatggaagcctgtgctcataacagtgcaaccttccccagtcagcacttcctcttcatggctttcccctaatgctatgcagtcaaccaatatattattgaattggggcaggtagaattaactcaattagattcttaagatcttttacaatggatgctattcattttctattctcatcagtgcatgcatttgacttgtagtgatgtttaaacctgaatagagtttaacagttgtggtaaggaacaattggtgggggagaaggaaggaatgttgtagacagggttgaagccacattgaagtaatatttatagtgaatgaattgttatacaatgagaataatagttatcgttatcttaaaagataaatttttaagtaaaacaaacatttctttctttgggtatcaaaaataaagagtaggctgggcatggtggtgcacgcctttaatcccagcactcaggagttcgaggccagcctggtctaattgtggaatatttcctaaaacatatgcagatgttttattcagtagacatctaatgaatttttgcatgctttttgatattgttatttgtacgtctttgatttcagtaagtggtgacttattggagatgtactttgagtcatgtatagtactagaaactaagaatataaataaagattgtccattcaccctctttacctgacaatacatgtttttaaaggaaaaatggctgatgaattgtaggatttttgtgataaatgatacagttgtaatttatgcaaaataaaaacatttacaagtatgacagaaatattccttagatccagtgatacaaaatgagactacacatctactttgtatataaatatttccatttggtttttgctttgctggggatcaaattcaatgtttcacacatattaggcaagcactctattagtgagctgaattcacaactcctggaggcacacaccatttataccagcctttaggcggtggaggcaggaggattaggagttcaaacccagactggattgcataaggcattaagtctagcctgagctacacagagagactctgtttcaacaaccaatggctggggacgtagctcagtagcagaatatatgcttagagtttacagagccctggggttggtcttcagcattataaaaaattaaaattagaattaaatgtacggtaaacatttaagtgaaagtctaaccactataatgacttcactgtttgcttatttaaaagtaagaaaatgacattgggaaacttgcaagttgattatttcttttgaagagctcctaaatgatcacaaatattttcaaaaatatttatgaattatgcagaactattttttttgagatatgatcttattgaaaaccttgggctggtttgtcactctgtgtaattaggctggtggtgaacttgagagtctcctgtctccccctcctggaactgtagacatgagctaccatgtctggcctacatgatgttaaaactgagaagaaaaagaaaagcaaagactttccattagaggaagcaacccaaaagacagttgggagtataagactgcaggaaatggagcatagttagattaaaagtattaaattaaggttggggtcagaagaggaagggccttggagaccattttaaatagctcgtactttgatctctgcatgttgcaatttagcagggtgttagtcctattatgtcacatctgatttaagttctgggggaaccacagtaatattaatgaggaggacaggttaaagtggtgcagactggaggctcgaggtccagtatggcttctcaaccttcttggcaagaaggctgcagggacgaccaggaagtttgaaacagtcttagaagaaaatgctggcttagagacaggtggcaatgggggatggggagcagtattctggtttgcatagaggcagagtccttccaagtgctgggaaacaaggcaggagggcagggatagagcaaatgatggctctgtatgtgtccctgttcagtttgcatttaatctgagcaaaatttggcttttgacatctgcaactcaaaagaaggtaattaggcaaatgactgacacatagatatcttaatagtcaaggaattttttttttttttttttttgaagagttagcagtcaggggatggtagaaactgcaaaaccaatccgtattctttcttgagatttttagacagttgatgctactagccacaaaaagagttttaagtgggaggagagtaagatgcaggcaccaaggtgacaggctccaggtctgtagcattagcttacagatgagattctttacagagagccaggcagctgcattggctaaagcagatctgggagggggccaggagatcagctggcggcactcccagcctccaggaaaggcaacccttatttctggaattttaaactgataacccaattcccaccagcctggccaggctcttccttagctcacatcacaaacacagaaggattgttttagatggagtcatgcttgattctttctatacctacttccaagaccaattttataaaagtttatttaccgccgtgtgtgtgtgtgtgtgtgtgtgtgtgtgtgtgtgtgtgtgtgtgtgcatggtatatatggacgtcagagtttggttctctccttctgcagtgtggctcttagagattgaactcagatcatgagcaagcaccttgctgcctgctatgtccctccagcagtctgaccatgttccttcccccaagattgtggaagctggactgaagatcacaatctgccagatgggcagaatctttactctttggcacatttgttgctgatggggagtgaatacccatggggacatggctgtcatggtgtggaagtgatagaaatgaaaacatgtatggatctgtcacaggagctggtgaggctgatgggtgtgtgggtggccactgtttgctctctgcttgtcacagcctcttgttcagggcttgatcagggaggtgtgtgtgtgtgtgtgtgtggtcacacccatctcagcagatctgtcagctttcccgcttttgttagagggtgatatcatgcttcctggggggagctctggaagacaatgagcagccactttcctctagatacaataggcggagtcaggaaggtagtattgacattgctggggcctaggagctactcactgctcggtggccgtcagatggtgaaccggcgtaaccttggcacacaggcctgggctgtacaaggcgtctggctgcagggccaaagaggactccaccctagggacaggagtacttcagacatctgggaatctgggatgggttttaaaattcagatcccaatataaaaaaacaactcccaaacaaacagcagcaattaaaaaaaaaaaaaaaaccagcctcccaagtaaaacaataatggtaccaggaataacaaagatcagtcacctaggtgctgagtcttttactatagctctcagcatcctgtctagtaggtattattcattttttaagtaccaaggcaaccaaggctcacatggctaaattacttgcccaaataggcatcaactatttggaacgtcctaacatatctatattcaaacgatgctaacccttctcactgtgactctgatgggactgtcgttcactggacacaaaatgtcttttctattgaaggacagcagacggttttacaaggatgatgcaaaatccaacaagagtttagaatcagcctgagacttttaatacaagatgtaagaagtaagatcttccccaaggtcagagttcttggccacttagggaagctggtatttaggaagagaactaggttgatagctgaagagaactctaacaggcacagatgttatttaacactggtccagtgccatgggaccagagatccatccctgtaacctgtaattacatggacttcattatttgtttggactgctttgtcctggtttctgtcttctgaaactgaagcattctgaatgatcgcatgcctccggggaaatggtggaaaacatgatttaagcaaagaatgacctaaacacacaaactttaaaacaagactaggtcacttcaggttgcatcctcaaaagcaaggacaacatgtgctttggggcaagggtttttaaagcacaactgcttaacagtacttgctacagtgtcagtatgtgataagcgtttaagtaatgggatggtcttaactgctttgtgtacaaagatggaccctcttatactgcctgatgctggtgtgttcagggcaggcctccattcctgagctaatctgtttatatatgtgtggggctgactctggctgctttgactgcaaaagagcatctttgtatcaggaaaacgaagagaagggcttaaagctttgcgttcctgggcttctgttaaaaagctgggttgtctggtggggaaattccttgcttgtgactcagccagccagcaaggtggagccccctacttttccccaaccaattcccaacttcttcttgcctctgggtcttgacttagcagaggttctaagatgacttgttgcttgtaactaccctccttatgttacttatcccaagtttgttgctacctaactggcagctgatcgaagcaagcagaagctgccacactttttttctactcatggaggtttgaatgtaggaccttacatatatatgcaggttgagatgtaaccagtgagctctacacccttgagagcctgtttccagtgtgattaagcttgttattatgtcataatgccacatgctcaatgcttatgtgttacaatagctctgctcacaacttgacacttctttctcctaatccctgcccccccccccatgtctgttctttgtgtccccaactcttgcatctgaaaagctcttagtactcttctaaggttgtttaatcatcctccgattttcatattcaggcctccaaggtcagaatcaaagagggagatggtggttgtgtttgaatctctttcattgccagtctttgcctactgactgcatcaaccggttggaactctcaggcctgcgtatgttggagctgtgctctgttcatgctgggttcctttcagtttctaaatatttacctttaagcctctgtgttagttgcattttgttggtcagtttttgtcagcttgacacaagctgaagctttctggggagaggaacctcaacagggaaaatacctgcattaaatttgcctataagcaaatctgagagacattttgttgatttaatgtttgatgtgggagggcccagtcccctgtgggtggccctactccaggggaggtggtcctggtgctataagaaaatagactgagaaacccaccaagaagtatctctccatggtctcttgagttcctgccccgacttccctcagcagtggactctaagtgtaagatgaaataaacactttcctctccaagttgttttggtcatggtgcttattacagcaatagaaacctgactctctctctctctctttccctctctctctctctctctctctctcacacacacacacacacacacacacacacacacacacacacacgacttatatatttgggtaaagcctggcattggaaaaggttcaaacacaaccaaccatgcatataaaatattctcttcctctgcttggagtaacctcctcacctgtcagacattggaagagaatccctactctatctaataagagcccataaatacatgtttgttgccatgaacaattctgggagatgacaggggtctcaccaagagggtggcatttgagaaggatgaataaccttttggaaaatcagtagatgtaaagagtagagggagatagtatagatggagagaaaagcattaaagtggaggataaaatgtttaagaaatacttcttcagttcctcagtgtgatgttactgtcacaacaatgaagaaagtgttagagtaaacattttgttagggaaaataggctaagaagaaggtaatttgtcttgagattggatgggaaggagacgcttatatggggccctttaggatatagttggaggccgtgagaagaatggaggtgttgcaggcctgtctggaaataaagaaaaaaagtaggtccaggaacacctggcagaaagagaaatctcaactctaatcatgtagctactggtaagttgcaggtactcctataaatagatgctttcttaggatcgagtcgacgccctatagtgagtcgtattagagctcgcggccgctctagaactagtggatcccccgggctgcaggaattcgatatcaagcttatcgataccgtcgacctcgagggggggcccggtacccaattcgctatagtgagtcgtattacgcgcgctcactggccgtcgttttacaacgtcgtgactgggaaaaccctggcgttacccaacttaatcgccttgcagcacatccccctttcgccagctggcgtaatagcgaagaggcccgcaccgatcgcccttcccaacagttgcgcagcctgaatggcgaatggaaattgtaagcgttaatattttgttaaaattcgcgttaaatttttgttaaatcagctcattttttaaccaataggccgaaatcggcaaaatcccttataaatcaaaagaatagaccgagatagggttgagtgttgttccagtttggaacaagagtccactattaaagaacgtggactccaacgtcaaagggcgaaaaaccgtctatcagggcgatggcccactacgtgaaccatcaccctaatcaagttttttggggtcgaggtgccgtaaagcactaaatcggaaccctaaagggagcccccgatttagagcttgacggggaaagccggcgaacgtggcgagaaaggaagggaagaaagcgaaaggagcgggcgctagggcgctggcaagtgtagcggtcacgctgcgcgtaaccaccacacccgccgcgcttaatgcgccgctacagggcgcgtcagccgtggcacttttcggggaaatgtgcgcggaacccctatttgtttatttttctaaatacattcaaatatgtatccgctcatgagacaataaccctgataaatgcttcaataatattgaaaaaggaagagtatgagtattcaacatttccgtgtcgcccttattcccttttttgcggcattttgccttcctgtttttgctcacccagaaacgctggtgaaagtaaaagatgctgaagatcagttgggtgcacgagtgggttacatcgaactggatctcaacagcggtaagatccttgagagttttcgccccgaagaacgttttccaatgatgagcacttttaaagttctgctatgtggcgcggtattatcccgtattgacgccgggcaagagcaactcggtcgccgcatacactattctcagaatgacttggttgagtactcaccagtcacagaaaagcatcttacggatggcatgacagtaagagaattatgcagtgctgccataaccatgagtgataacactgcggccaacttacttctgacaacgatcggaggaccgaaggagctaaccgcttttttgcacaacatgggggatcatgtaactcgccttgatcgttgggaaccggagctgaatgaagccataccaaacgacgagcgtgacaccacgatgcctgtagcaatggcaacaacgttgcgcaaactattaactggcgaactacttactctagcttcccggcaacaattaatagactggatggaggcggataaagttgcaggaccacttctgcgctcggcccttccggctggctggtttattgctgataaatctggagccggtgagcgtgggtctcgcggtatcattgcagcactggggccagatggtaagccctcccgtatcgtagttatctacacgacggggagtcaggcaactatggatgaacgaaatagacagatcgctgagataggtgcctcactgattaagcattggtaactgtcagaccaagtttactcatatatactttagattgatttaaaacttcatttttaatttaaaaggatctaggtgaagatcctttttgataatctcatgaccaaaatcccttaacgtgagttttcgttccactgagcgtcagaccccgtagaaaagatcaaaggatcttcttgagatcctttttttctgcgcgtaatctgctgcttgcaaacaaaaaaaccaccgctaccagcggtggtttgtttgccggatcaagagctaccaactctttttccgaaggtaactggcttcagcagagcgcagataccaaatactgtccttctagtgtagccgtagttaggccaccacttcaagaactctgtagcaccgcctacatacctcgctctgctaatcctgttaccagtggctgctgccagtggcgataagtcgtgtcttaccgggttggactcaagacgatagttaccggataaggcgcagcggtcgggctgaacggggggttcgtgcacacagcccagcttggagcgaacgacctacaccgaactgagatacctacagcgtgagctatgagaaagcgccacgcttcccgaagggagaaaggcggacaggtatccggtaagcggcagggtcggaacaggagagcgcacgagggagcttccagggggaaacgcctggtatctttatagtcctgtcgggtttcgccacctctgacttgagcgtcgatttttgtgatgctcgtcaggggggcggagcctatggaaaaacgccagcaacgcggcctttttacggttcctggccttttgctggccttttgctcacatgttctttcctgcgttatcccctgattctgtggataaccgtattaccgcctttgagtgagctgataccgctcgccgcagccgaacgaccgagcgcagcgagtcagtgagcgaggaagcggaagagcgcccaatacgcaaaccgcctctccccgcgcgttggccgattcattaatgcagctggcacgacaggtttcccgactggaaagcgggcagtgagcgcaacgcaattaatgtgagttagctcactcattaggcaccccaggctttacactttatgcttccggctcgtatgttgtgtggaattgtgagcggataacaatttcacacaggaaacagctatgaccatgattacgccaagcgcgcaattaaccctcactaaagggaacaaaagctggagctccaccgcggtggcggccgc
